# Supplementary material for: Orientation-invariant autoencoders learn robust representations for shape profiling of cells and organelles
Source: Nat Commun. 2024 Feb 3;15:1022. doi: 10.1038/s41467-024-45362-4 (PMC10838319; doi:10.1038/s41467-024-45362-4)
Supplement: Supplementary file 1 — Supplementary Information [file 41467_2024_45362_MOESM1_ESM.pdf]

Supplementary Information

"Orientation-invariant autoencoders learn robust representations for shape profiling of cells and organelles"

Supplementary Note 1

This supplementary section supports the second results section and the relevant Methods sections. The topics are: image orientation, pre-alignment, and their impact on embedding accuracy.

Supplementary Note 1a: Workflow of standard pre-alignment algorithms

See Methods for procedure details and citations. In the below figure, all images (left box) are rotated so that its 'major axis' aligns with the y-axis (second box). Since the object can be flipped in the x- or y-axes or both, there are four valid flips that still have aligned major axes. To resolve this, each of the four flips is computed (third box), and the 'best flip alignment' is chosen as the final orientation (right box). The criteria for 'best flip alignment' is having highest cross-correlation with a reference image. and the reference image is the mean image in pixel space (bottom box). The flip-alignment stage is repeated until the cross-correlation with the mean image converges (corresponding to a consistent set of flips), which in our experiments took fewer than 30 iterations.

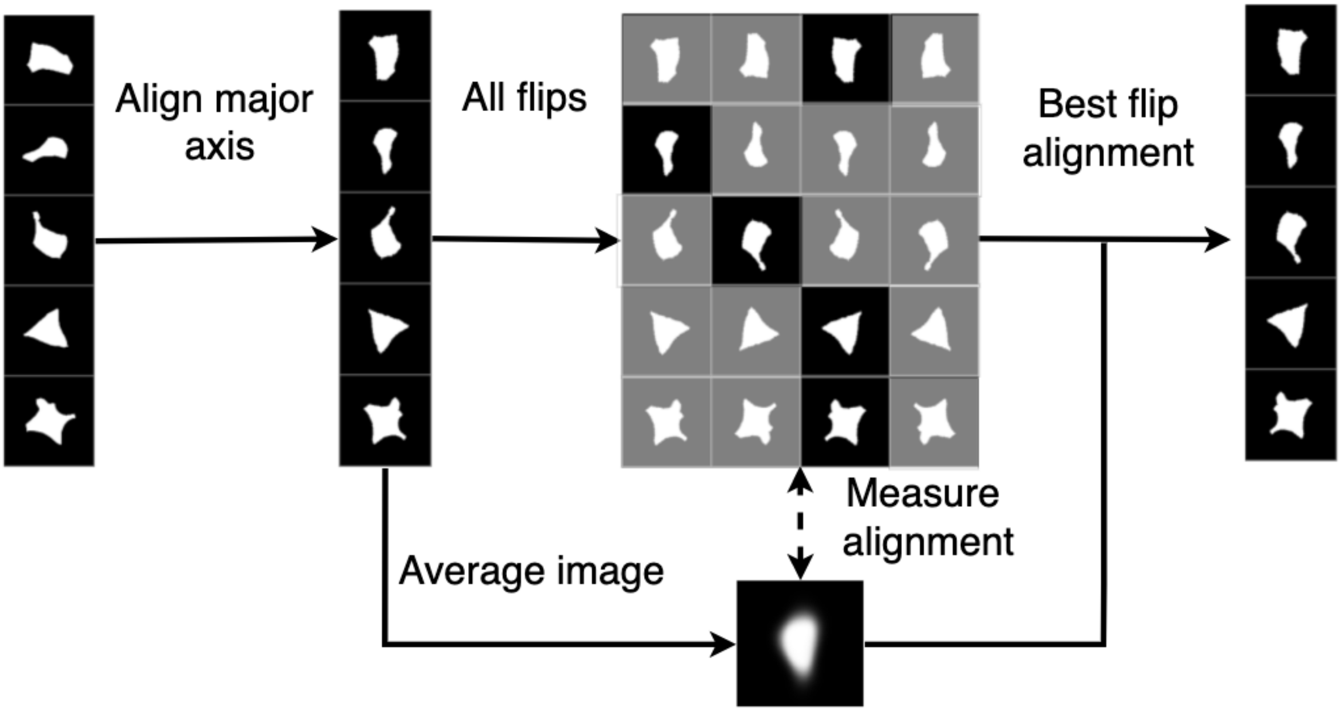

Supplementary Fig 1: Workflow of standard pre-alignment algorithms The workflow for doing pre-alignment, as described in detail in the text above the figure.

Supplementary Note 1b: Embedding error margin for varying threshold

The 'embedding error' score in Fig.2c-d (from the main text) depends on a choice of threshold, which we chose as t=100. In Supplementary Fig 2, we plot the embedding error for O2-VAE and prealign-VAE at many choices of `k` and for each cell line. This shows that prealign-VAE has more embedding errors than O2-VAE for a wide range of choices for t, so the Fig.2c-d results (from the main text) are not an artefact of the choice of t.

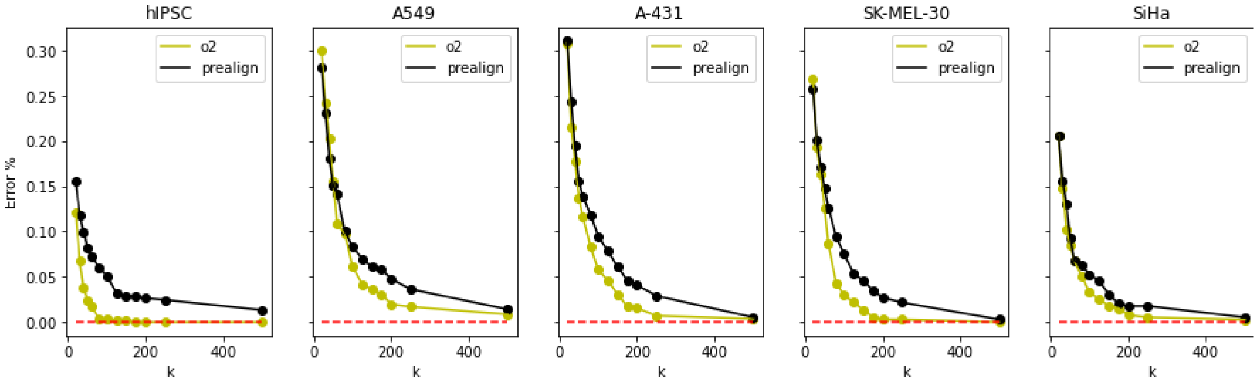

Supplementary Fig 2: embedding error margin for varying threshold.
Embedding error margin for varying threshold for 5 cell lines: hIPSC from [1] and four HPA lines [17]: A549, A431, SK-MEL-30 and SiHa. The y-axis is embedding error rates. The x-axis is an integer threshold parameter, k, which we now explain. Given a pair of cell images that are 'very similar' (as defined in "Methods - Quantifying embedding errors"), take their embeddings. If the pair of embeddings are not k-nearest neighbours, it is an embedding error.

Supplementary Note 1c: Supporting discussion on pre-alignment errors cause embedding errors, and it is difficult to fix them

We first investigate embedding errors for prealign-VAE and establish that they are likely caused by pre-alignment errors.

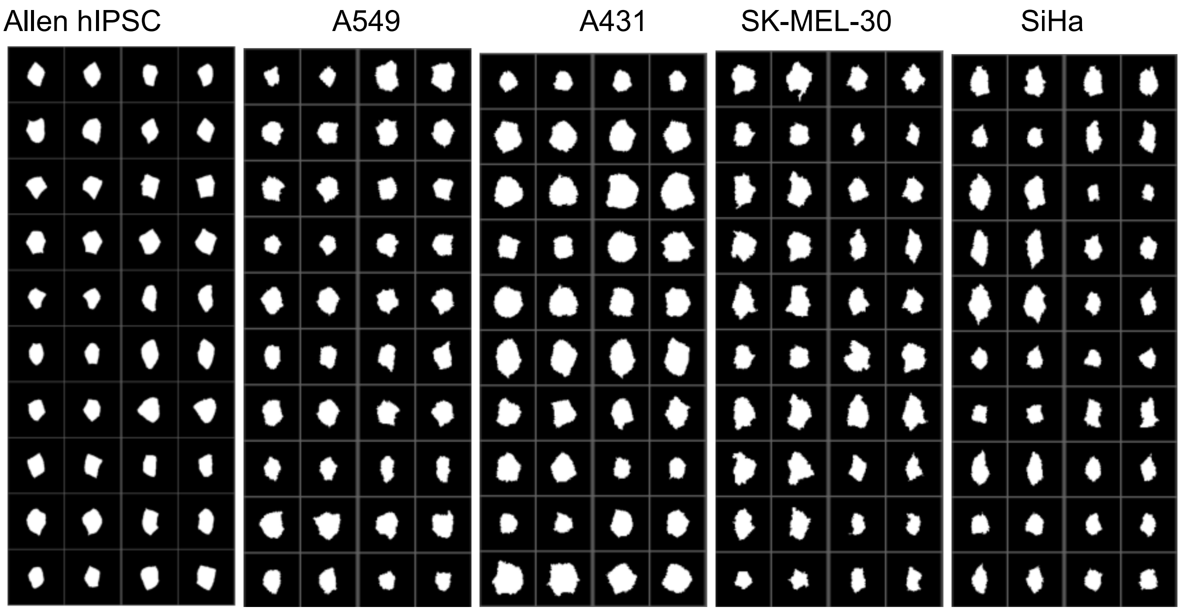

Supplementary Fig 3: pre-alignment errors explain the gap in prealign-VAE vs O2 error rates. For each test dataset (Allen hiPSC and four human protein atlas cell lines), this displays sample image pairs that are embedding errors for prealign-VAE but not O2-VAE. For each, column 1 and 2 are pairs, and column 3 and 4 are pairs.

In Results, we claimed that prealign-VAE has higher embedding errors because of image pairs having bad alignments, and that is supported by Supplementary Fig 3. Most of these pairs are similar in shape, but they are poorly aligned with each other. Only a minority of these pairs are actually well aligned.

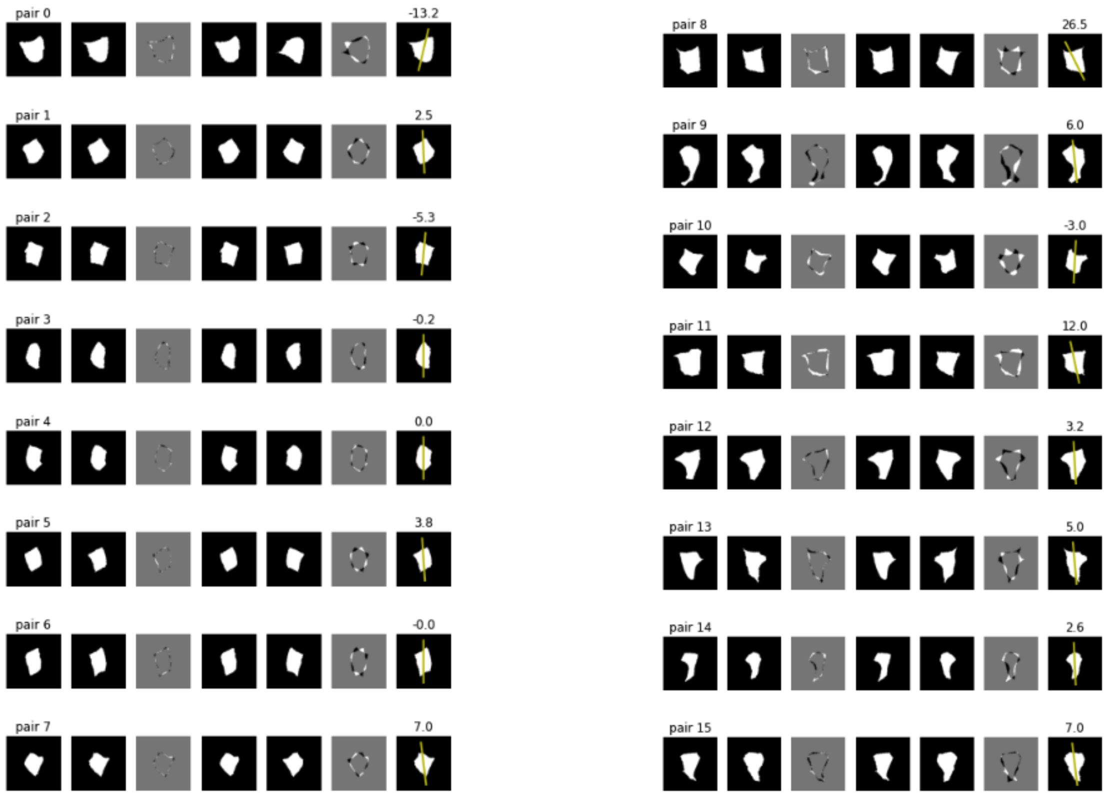

Supplementary Fig 4: Embedding errors and their issues with pre-alignment
Investigating 16 example pairs from the hiPSC dataset that are “embedding errors” for the prealign-VAE (which is just a regular VAE where images are pre-aligned, as explained at the start of this Supplementary). Each row is a pair and labeled with an index between 0 and 15 above the first image in the row. The 1st and 2nd columns are the two objects after aligning them optimally, and the 3rd column is the residual plot (1st column minus 2nd column in pixel space). The 4th and 5th columns show the images in their orientation after applying the pre-alignment algorithm, and the 6th column is their residual (4th column minus 5th column in pixel space). The 7th column shows the second object in its optimal alignment with respect to the first object. For the 7th column, we also draw a line corresponding to its major axis and write the angle between this and the y-axis above the image. Since the first object has its major-axis on the y-axis, this also measures the angle between first and second object major axes when they are aligned with each other.

Before explaining insights from this figure, we need one more figure:

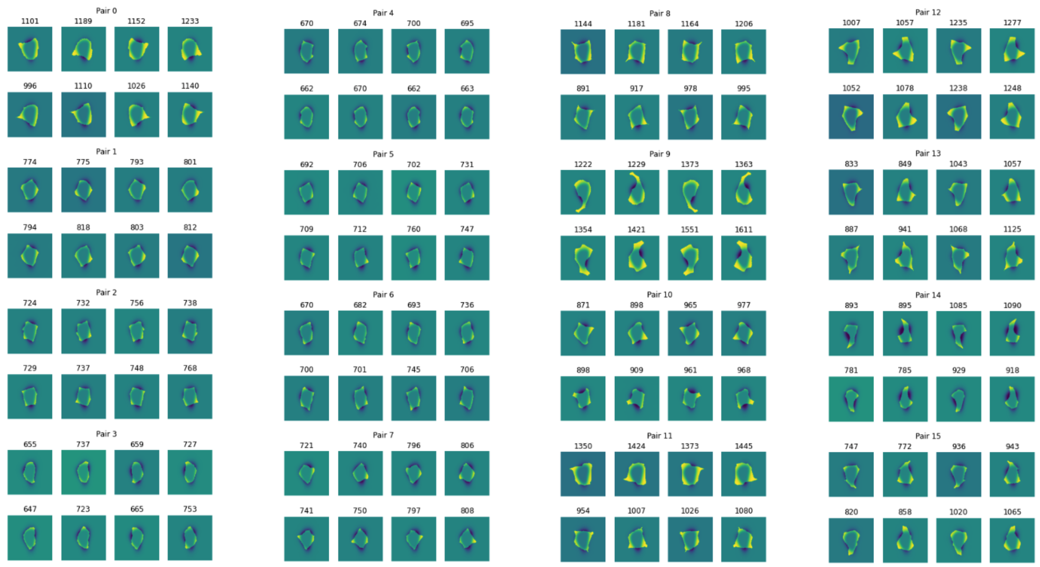

Supplementary Fig 5: flip alignment decision for images with embedding errors. The pre-alignment procedure aligns the major-axis and then flips the image to best align with a reference cell (the reference cell is the image mean in the dataset). Above, each row is one of the images from Supplementary Fig 3 in each of the four possible flip orientations. The image shows the residual compared to the mean cell, and the number above is the absolute pixel error over the image. The 1st column of each row is the orientation with minimal pixel error - this is the orientation chosen by the pre-alignment algorithm.

The goal of pre-alignment is to ensure that any image pairs with similar shapes (like all these examples) will be oriented in the same way. For these embedding errors, it's clear that most of the images after pre-alignment are mis-aligned with each other. Supplementary Fig 3 shows this: columns 4 and 5 are the pre-aligned objects, and column 7 shows the orientation of the second image if properly aligned with the first image.

Failure modes of pre-alignment

Supplementary Figs 4 and 5 highlight two major failure cases.

Case 1 is *major-axis mis-alignment*. The objects in their optimal alignment have different major-axis angles. In Supplementary Fig 5, column 4 is the first object after pre-alignment, and column 7 is the second object aligned with the first object with its major axis annotated. This failure case has the major axis that is far from the y-axis (example images 0, 7, 8). The best pairwise alignment is therefore not possible because pre-alignment always forces the major axis to be aligned with the major axis. So this constraint makes pre-alignment computationally tractable, but also introduces this failure case.

Case 2 is *unstable flip alignment*. Image orientation is unstable in the sense that the pre-aligned image and its flipped version have very similar cross-correlation with respect to the mean cell. A slightly perturbed version of the same image may therefore be flipped by the pre-alignment

procedure. All examples above that are not case 1 (i.e. examples with a small angle in column 7) are examples of unstable flip. Supplementary Fig 5 shows that flipped versions of the image have very similar error. This is the more common case.

Commentary

One natural approach is to add new heuristics to the pre-alignment algorithm to address the 2 failure modes just described.

We experimented with changes to the pre-alignment algorithm to address the failure case 2. We hypothesised that some objects have similar flip cross-correlations with the mean cell because they are very small (or very large) compared to the mean cell. The reasoning was that for very small (or large) cells, the contour details of the cell were fully contained (or fully outside the bounds of) the mean cell, so the correlation error would not change when flopping. The solution was to scale-normalise all objects before doing flip-alignment.

But this modified version of pre-alignment still had failure cases. This suggested that finding heuristic fixes to pre-alignment is very challenging. Heuristic fixes that help with one dataset may not help with another one. We hypothesise that it is difficult to find a pre-alignment algorithm that is simple, while working robustly across many datasets.

Looking into the failure cases specifically, Failure case 2 appears because the dataset is diverse, so there is no single image that can serve as a good reference for all input images. Failure case 1 is very difficult to overcome without a complete change of approach. The choice of aligning the major axis with the y-axis constrains the search space of image orientations. If we don't do this constraint, the optimization would become computationally intractable.

In summary, global pre-alignment is hard because (i) major axis alignment - which is necessary to make the algorithm computationally tractable - causes some similar pairs to be misaligned; and (ii) it is difficult to find a good reference shape to do registration for a diverse dataset. We claim that these issues are very hard to overcome.

Finally we make the point that these errors are present for segmentations of cells, which is a simple setting (the only simpler one would be segmentation of nuclei). In settings, such as joint cell and nucleus segmentations or grayscale images, pre-alignment is probably even more challenging.

Supplementary Note 1d: Methods for finding similar-object-pairs in embedding error experiments

First we justify the similarity metric proposed in the Methods, especially the fact that we raised the normalizer to the power of 1/2. We want a metric that can identify pairs that are very likely to have similar shape. The RMSE is one approximation:

$$RMSE(x,y) = \left(\frac{1}{m \cdot n} \sum_{i,j} (x_{i,j} - x_{i,j})^2 \right) \tag{1}$$

But we observe that if the scale of an object increases, the same (relative) perturbation will cause more pixels to change, increasing this score. In RMSE, the sum of squared differences is scaled by the number of image pixels: $m \cdot n$. To avoid the scale bias, we replace this scalar by the size of the image. For binary masks, this is the number of nonzero pixels, which we write with the function $s(\cdot)$. We scale by whichever image is largest: $max\{s(x), s(y)\}$.
Replacing the scaling terms $m \cdot n$ with $max\{s(x), s(y)\}$ is mathematically equivalent to dividing the RMSE with by the scalar:

$$C = \left(\frac{m \cdot n}{max\{s(x), s(y)\}} \right)^{\frac{1}{2}} \tag{2}$$

And dividing by this scalar gives the $NRMSE$ introduced in the Methods. (Actually we remove the $\sqrt{m \cdot n}$ term since this is the same for all images, and so it does not affect the relative ordering of $NRMSE$ scores, which is all we need).

Next, we show evidence that "high-confidence-similar-pairs" really are semantically similar with the below figure.

(a)

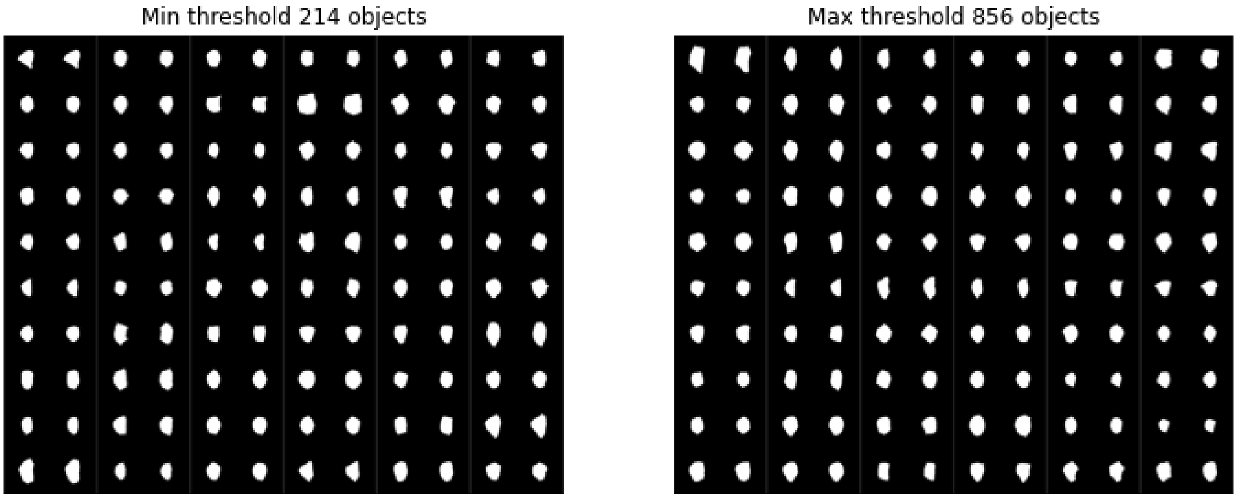

(b)

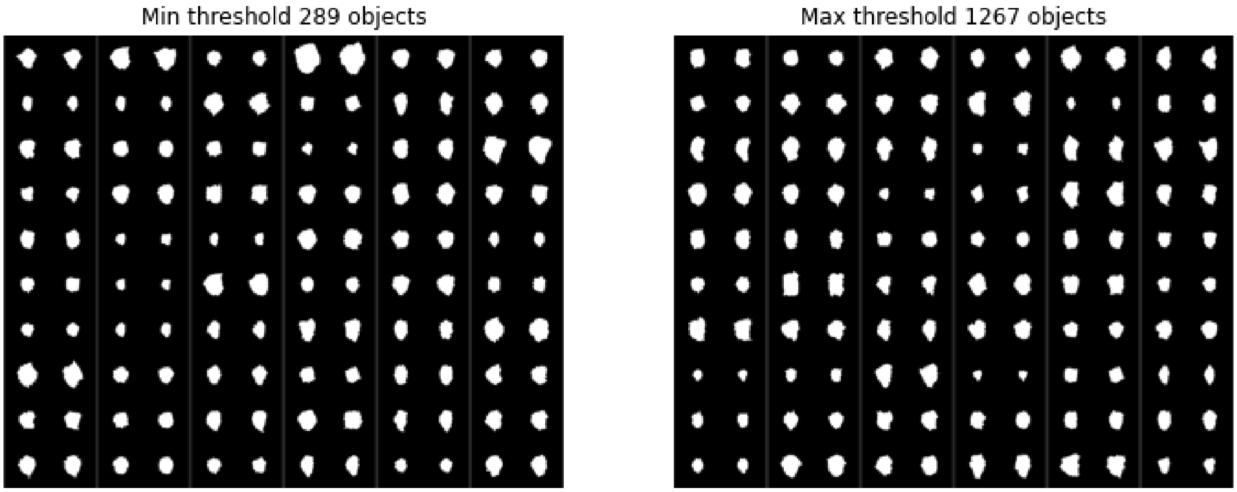
Min threshold 289 objects
Max threshold 1267 objects

(c)

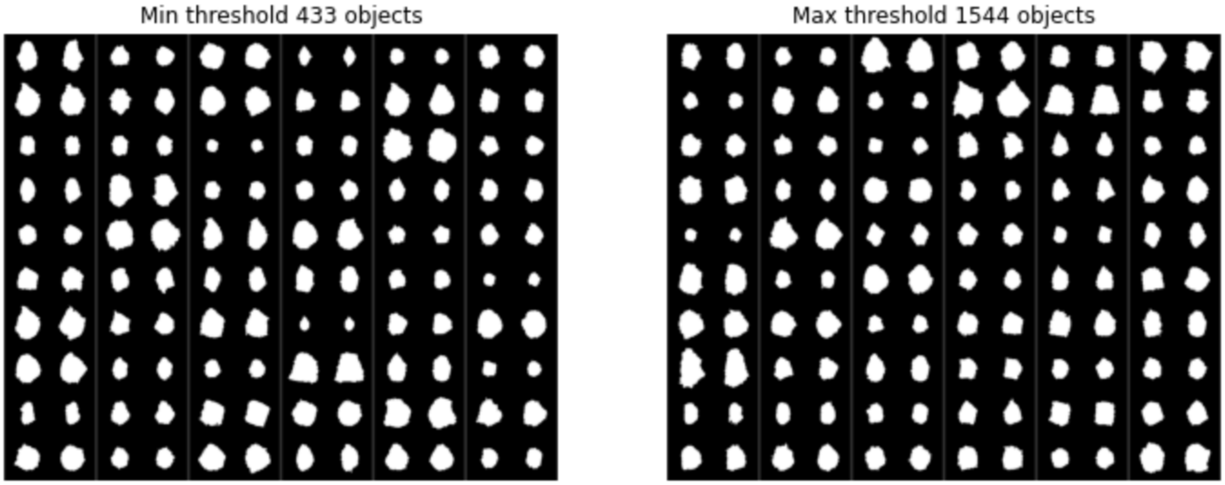
Min threshold 433 objects
Max threshold 1544 objects

(d)

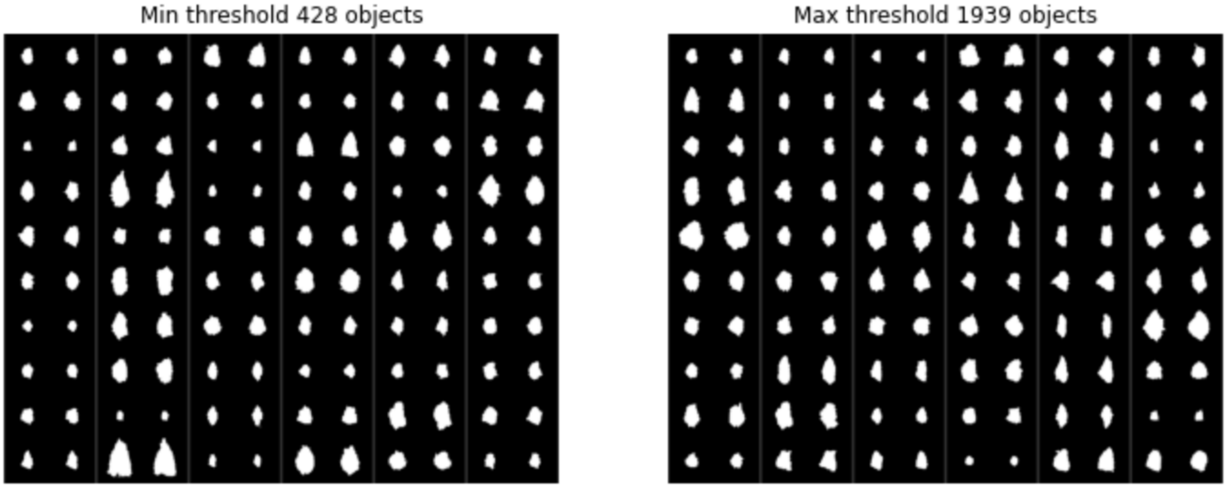
Min threshold 428 objects
Max threshold 1939 objects

Supplementary Fig 6: Sample 'high-confidence-similar-pairs' for embedding error experiments. A random sampling of pairs thresholded to be 'similar with high confidence' (column 1 and 2 are pairs, column 3 and 5 are pairs, etc). In experiments we try a range of thresholds, so for each dataset below, the left plot is a sample of images when using the smallest threshold, and the right plot is using the largest threshold. They are plotted in the orientation after doing pre-alignment. Cell lines are: **a** hiPSCs, **b** HPA A 549, **c** HPA A 431, **d** SK MEL 30.
As discussed in Methods, this approach for identifying semantically-similar objects is approximate. We aim only to have a low false positive rate (which is what the figures show), but we cannot guarantee a low false negative rate.

Supplementary Note 1e: O2-VAE embedding errors

Why does O2-VAE still have embedding errors? We investigate with the following figure.

a

Allen hIPSC A549 A431 SK-MEL-30 SiHa

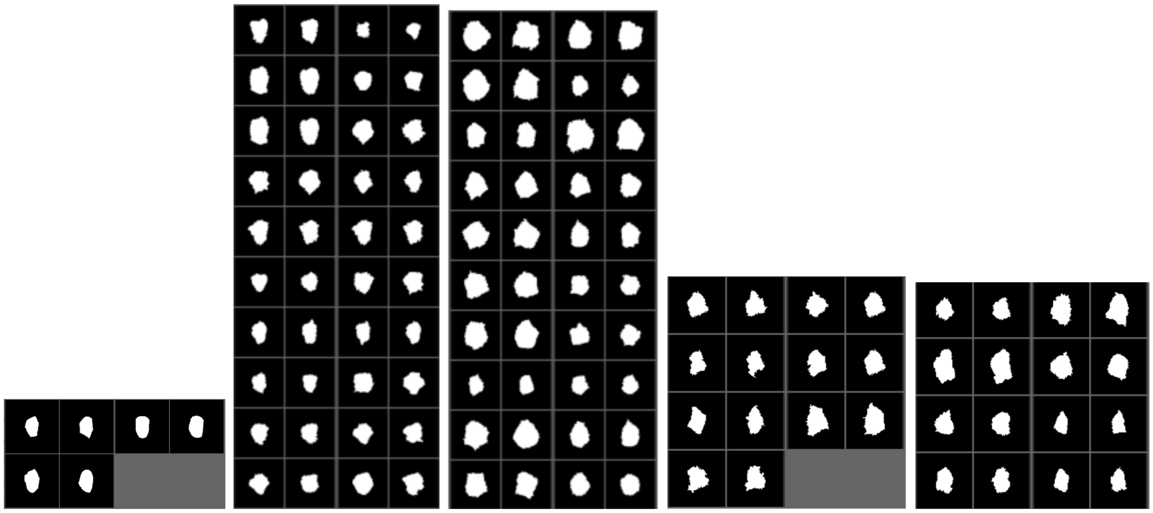

b

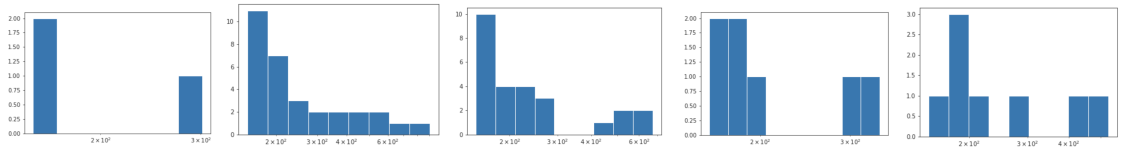

c

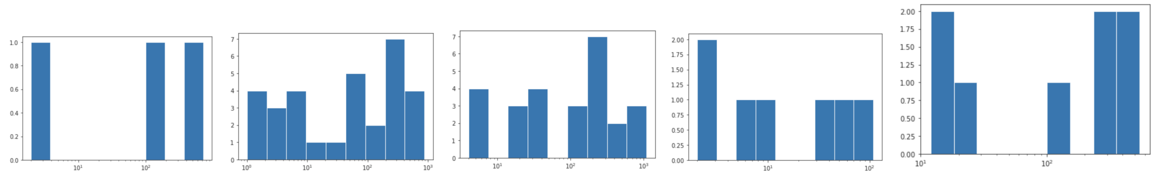

Supplementary Fig 7: Investigating the cause of O2-VAE embedding errors. 'High-confidence-image-pairs' where O2-VAE had embedding errors for the threshold $t = 100$ that was chosen for experiments. **a.** Sample image pairs. We show either 20 pairs (for two cell lines), or all of the pairs where there were fewer than 20 errors (for three cell lines). **b.**

Histogram of the ‘kNN distance’ between the images on a log scale, so that we can tell how far above threshold these image pairs are. **c.** The same histogram as b of the ‘kNN distance’, but for the prealign-VAE.

Discussion:

- Firstly note that for 3 of the datasets, there are very few examples: fewer than the 20, so O2-VAE makes very few embedding errors.
- One explanation is that the images may not actually be that visually similar. The definition of “similar” is pixel-based and based on a threshold, so you would expect a few images that are not actually semantically similar. In that case, it’s reasonable for the embedding distance to be larger.
    - One piece of evidence supporting this is to also look at the `k` distribution histograms for prealign-VAE. For some datasets, the distribution of `k` for prealign-VAE is wider. (The cases where this was not true were dataset having very few instances of O2 errors.).
    - But there are few such examples, suggesting that the ‘similar image pair’ metric does achieve the desideratum of having low false positives rate.
- The second explanation is that the images are still close, but the kNN distance is slightly above the threshold. This is also supported by looking at the histogram of kNN values.

Supplementary Note 2

This supplementary supports the third Results section.

Supplementary Note 2a: Synthetic shape dataset: PCA of embedding space classes

In Fig.3.a (from the main text) we showed samples from the synthetic shape dataset, a distance matrix between class centroids and UMAPs. We used these to claim that eccentricity variation was the dominant factor of variation followed by contour randomness. Below, Supplementary Fig8 shows the same thing. Eccentricity dominates in PC1, and randomness dominates in PC3.

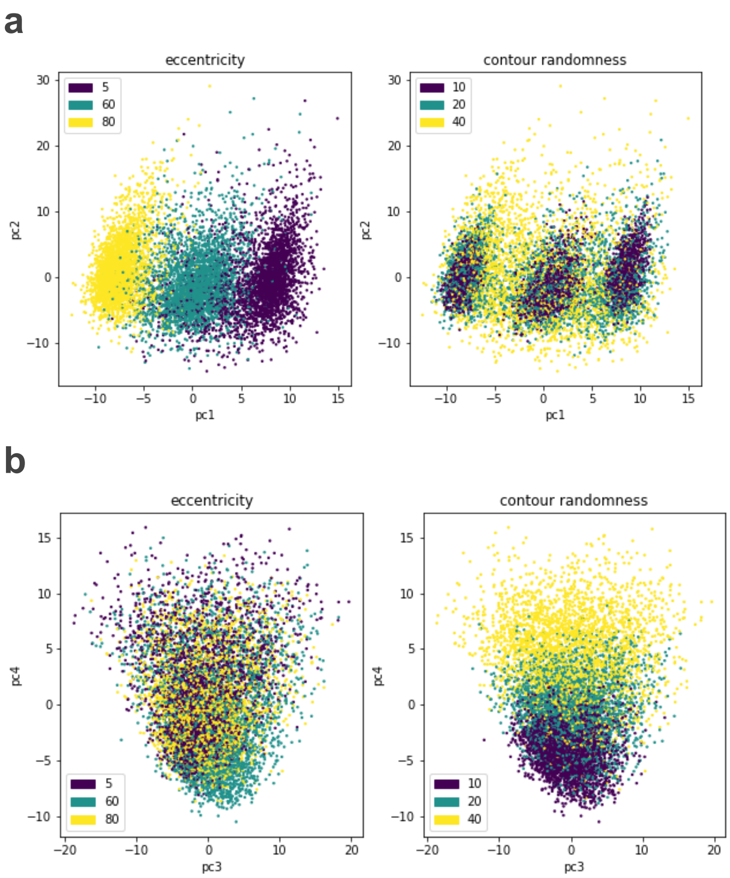

Supplementary Fig 8: PCA of synthetic shape dataset coloured by generative factors
a. Projection to the plane with PC1 (x-axis) and PC2 (y-axis) coloured by eccentricity (left) and randomness (right). **b.** the same, except projection on the plane of PC3 and PC4.

Supplementary Note 2b: Synthetic shape dataset: class overlap for high randomness classes

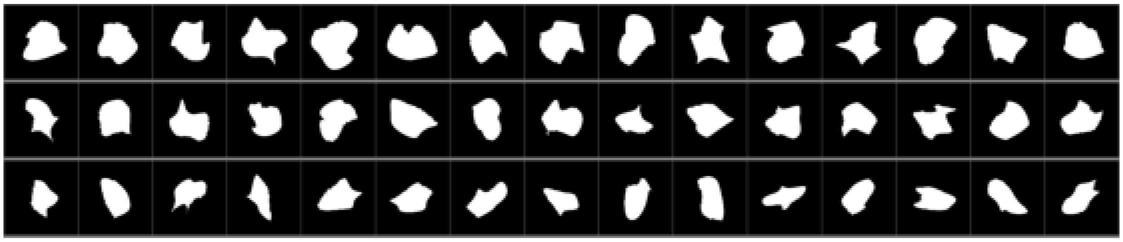

Supplementary Fig 9: image samples for high random classes
Each row is random samples from each of the three ‘high random’ data classes. The classes are ordered from highest to lowest eccentricity.

In the third Results section, we claimed that for high randomness classes, the ‘boundary’ between classes with different eccentricity can be overlapping. This can be seen by observing that some samples between classes are very similar, for example the first two rows of the second column. This is a result of the sampled generative process.

The embedding space dimensionality reduction plots also indicate that the high randomness classes are overlapping: UMAP in Fig 3a and PCAs in Supplementary Note 2a. Since Supplementary Fig.9 shows that the classes really do overlap, we can see that this is likely a feature of the data, and not an issue with the learned representations failing to separate classes.

Supplementary Note 2c: Verification tests: comprehensive and quantitative orientation invariance test

We do a more direct and comprehensive test for orientation invariance, and in figures below, we visualise what these metrics mean

- For each object in the test set, take a set of rotations and flips (figure left panel), and compute their representations.
- The representations should not change, but due to discretisation artefacts, the output encodings will change a bit (see Methods discussion). We want to validate that the difference is small. Figure top-right is a histogram of the distances between encodings of different orientations. We measure the *biggest* embedding distance of the same image due to rotations and flips (the biggest value in Figure top right).
- We measure the embedding distance between the original image and *every other image* in the dataset (the histogram of these values is figure bottom right). We identify the *smallest* such distance, which is the 1-nearest neighbour (we mark this as a red line in figure bottom-right).
- We check that the *biggest* encoding distance due to rotations and flips is smaller than the *smallest* encoding distance between other images in the dataset. The top and bottom of the figure have the same x-axis, which shows that this condition is met.

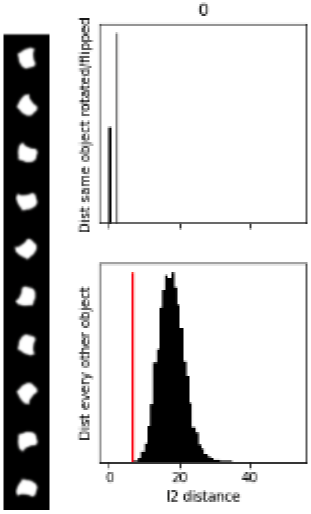

Supplementary Fig 10: visualisation of the metrics for quantitative orientation invariance test. (Left) ten orientations of the same image for testing: five rotation sets for two flips. We take the embeddings for all these images. (Right, top) histogram of the distance matrix values between all ten embeddings. (Right bottom) histogram of the distances between the original image embeddings and all other values, with a red line marking the smallest value.

In Supplementary Fig 10 (right, bottom), if we have orientation invariance, the red line should be at a larger value than all of the points in the top histogram. Our invariance test passes for every object in the Allen hiPSC dataset.

Supplementary Note 2d: hiPSC data: extended analysis figures Allen hiPSC cells and nuclei

In the third results section and Fig.3b, we visualise the shape variation in the Allen hiPSC dataset after scale-normalisation. We did clustering, and now show another summarisation approach: dimensionality reduction

a

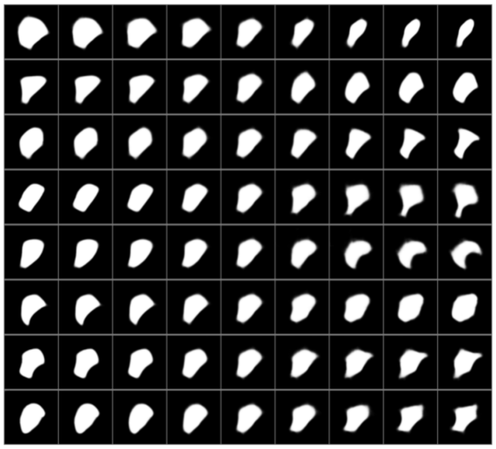

b

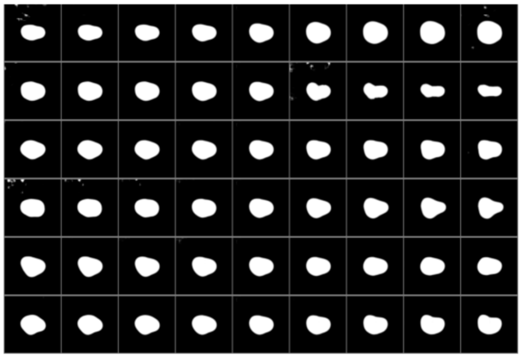

Supplementary Fig 11: Allen hiPSC scaled data - PCA traversals. a. PCA traversals of scale-normalised Allen hiPSC cells. Each image is a point in the representation space visualised by passing that representation through the decoder. Row i is the i'th PC. The mid-point is the origin, and the images in the rest of the row are equally spaced points between -2σ and 2σ where σ is the standard deviation of the representations along that PC. **b.** The same PCA traversal plot for the nucleus.

a

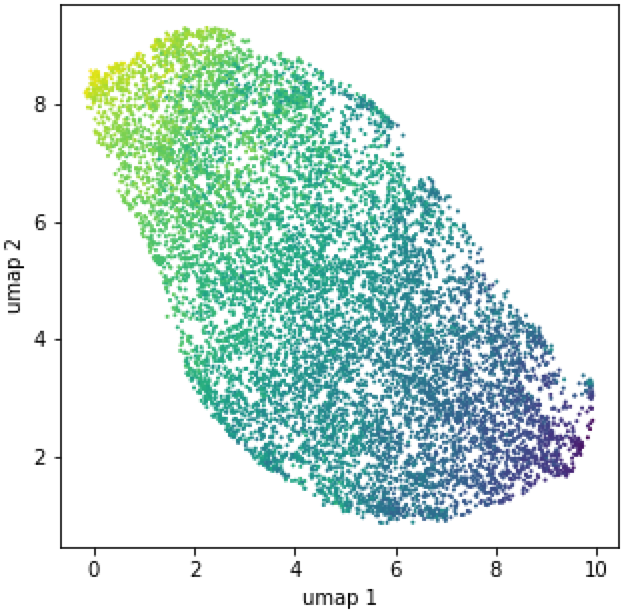

b

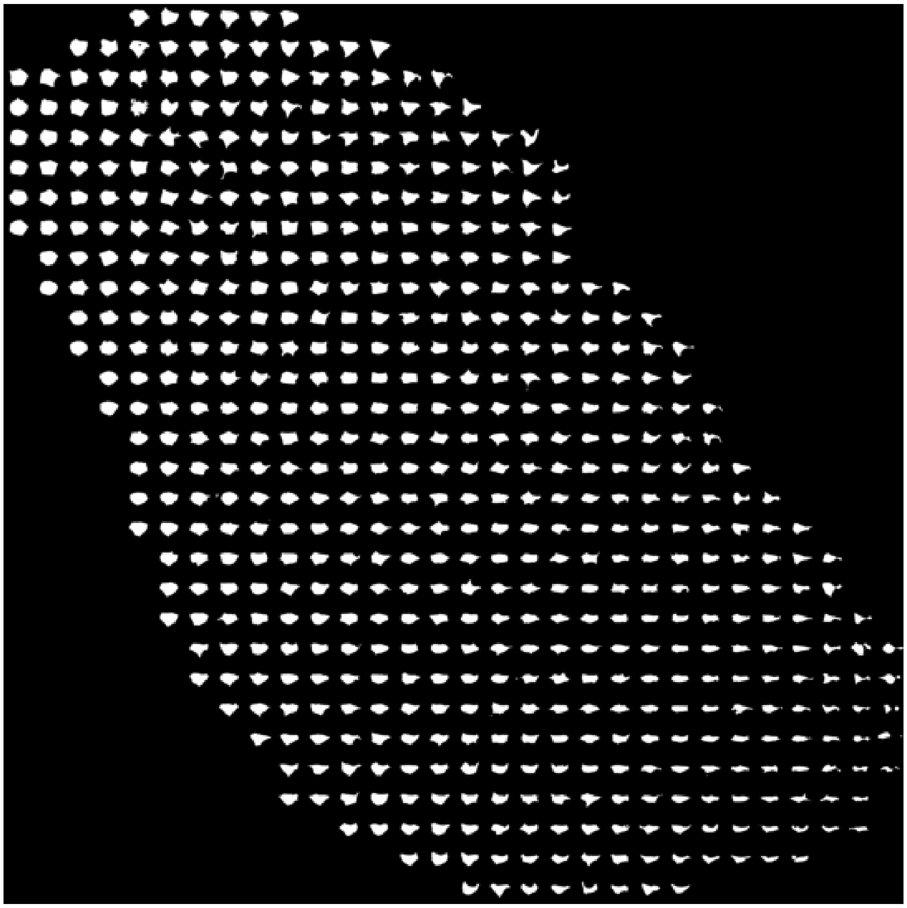

Supplementary Fig 12: Allen hiPSC scaled data - UMAP representation. a. Scatterplot of UMAP-reduced Allen cell hiPSC cell data (scaled). The colouring is with respect to area. **b.** The same UMAP data but we sample images from the real dataset. We create a grid in the UMAP space and sample the image whose reduced embedding is nearest the centroid.

Supplementary Note 2e: hiPSC extended data: visualising shape variation for non-scaled cellular data

In the third results section and Fig.3b, we visualise the shape variation in the Allen hiPSC dataset after scale-normalisation. Here we do clustering and dimensionality reduction of cells without scale normalisation

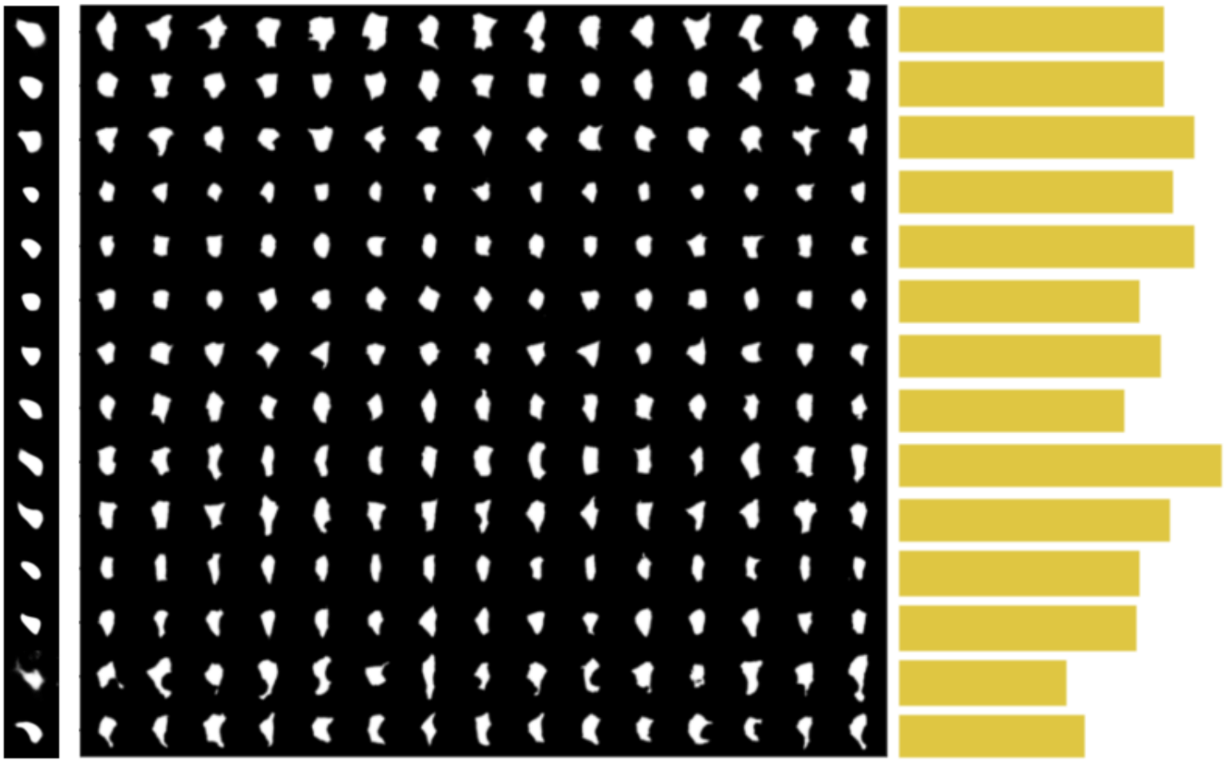

Supplementary Fig 13: clustering of Allen hiPSCs without scale normalisation
a Cell clustering with GMM and k=14. Prototypes (left), cluster samples (middle) and population frequencies (right).

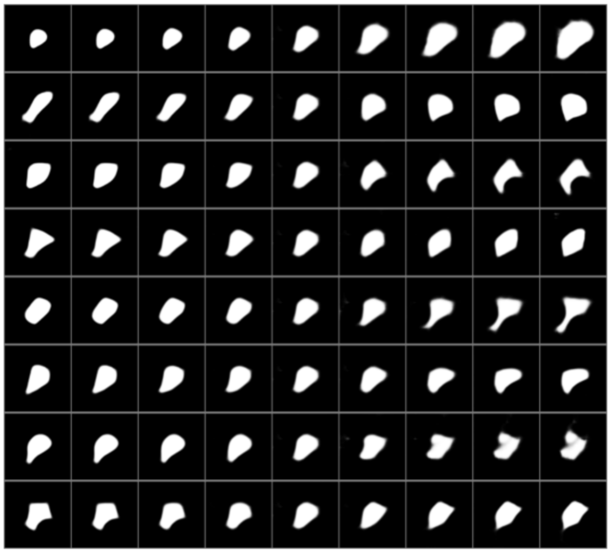

Supplementary Fig 14: Allen hiPSC non-data - PCA traversals

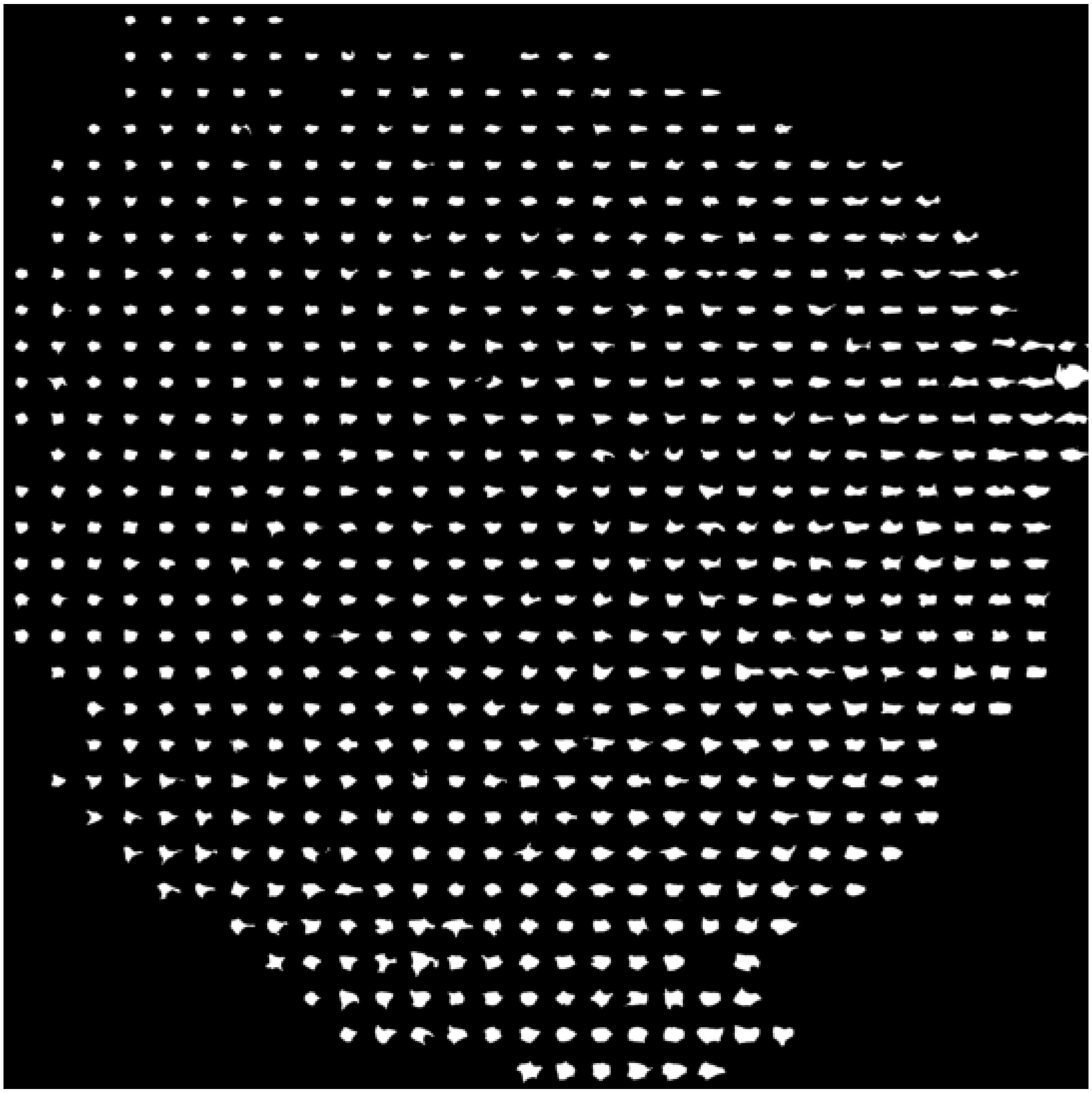

Supplementary Fig 15: Allen hiPSC non-scaled cells - UMAP representation
UMAP-reduced data we sample images from the real dataset.

Supplementary Note 2f: Clustering performance of o2vae vs baseline autoencoders on Allen hIPS dataset

We repeat the clustering analysis from Figure.3c. The dataset is Allen hIPSC cells [1], which are scale-normalised based on major axis length. This is a real dataset without ground-truth class labels, so evaluation must be qualitative. We do GMM clustering with K=8. Each row shows random samples from each cluster in their original orientation, since orientation can be a confounder.

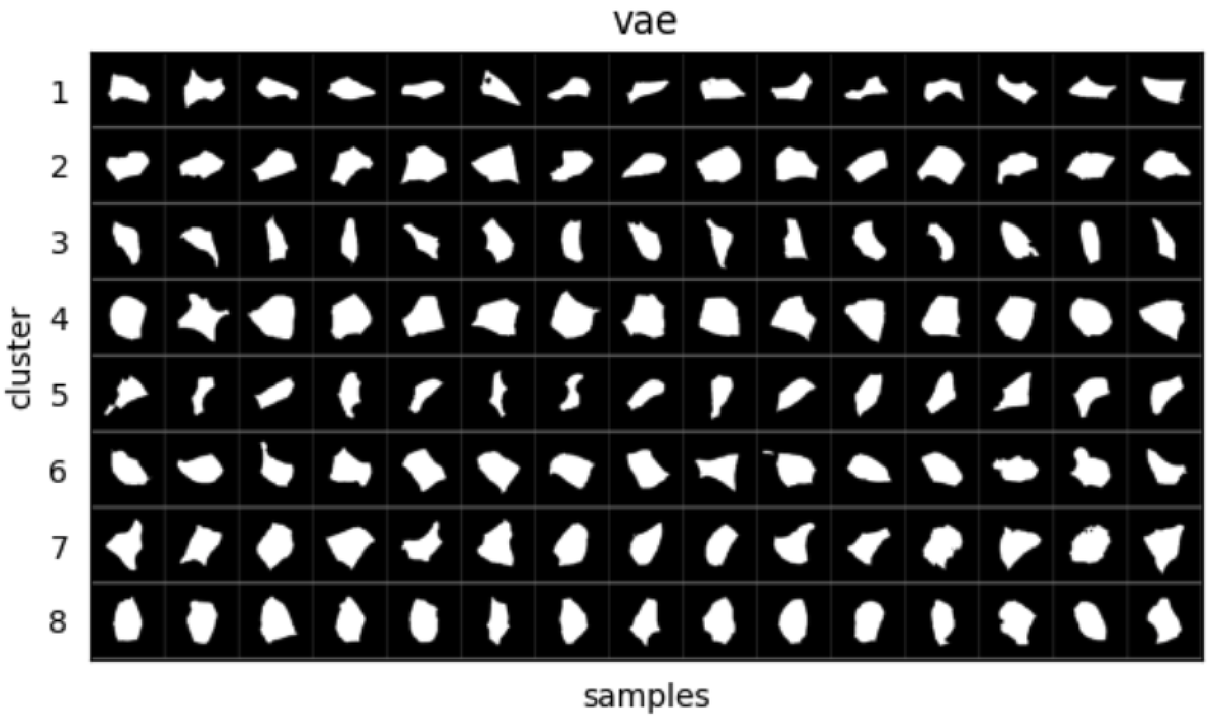

Supplementary Fig 16: Allen hiPSC scaled cells - clustering of VAE representations. GMM clustering over VAE representations with k=8 for hiPSC cells.

Comments:

- An ideal clustering would have groups of similar shape, regardless of orientation.
- But the orientation (or pose) is a confounding variable. Clusters 1 and 2 are mostly cells with horizontal orientation, while clusters 3 and 8 have vertical orientation. For example, there are cells in clusters 1 and 3 with similar shape, but they have different clusters.

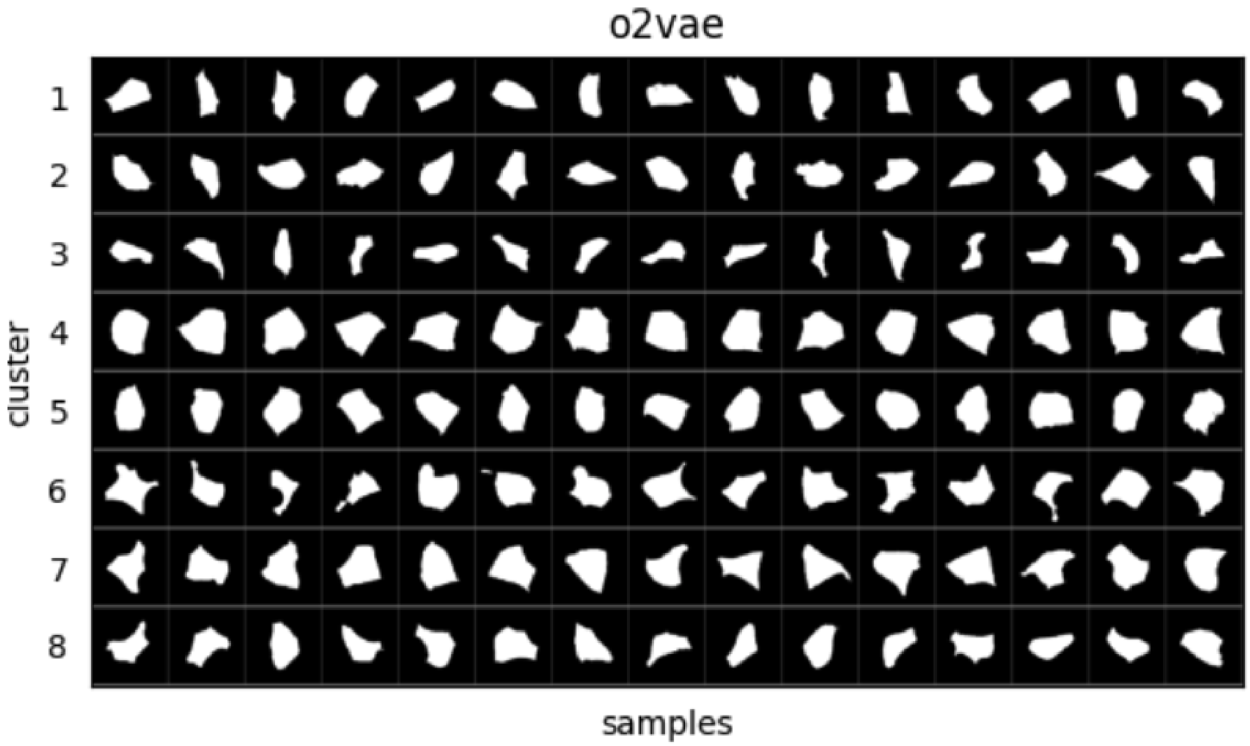

Supplementary Fig 17: Allen hiPSC scaled cells - clustering of o2-VAE representations. GMM clustering over O2-VAE representations with k=8 for hiPSC cells.

Comments:

- Unlike in VAE, orientation seems not to be a confounder.
- (NB: this figure is different from Fig.3c because of the different random seed used in clustering, so the order of rows changes; but the morphology groups are similar).

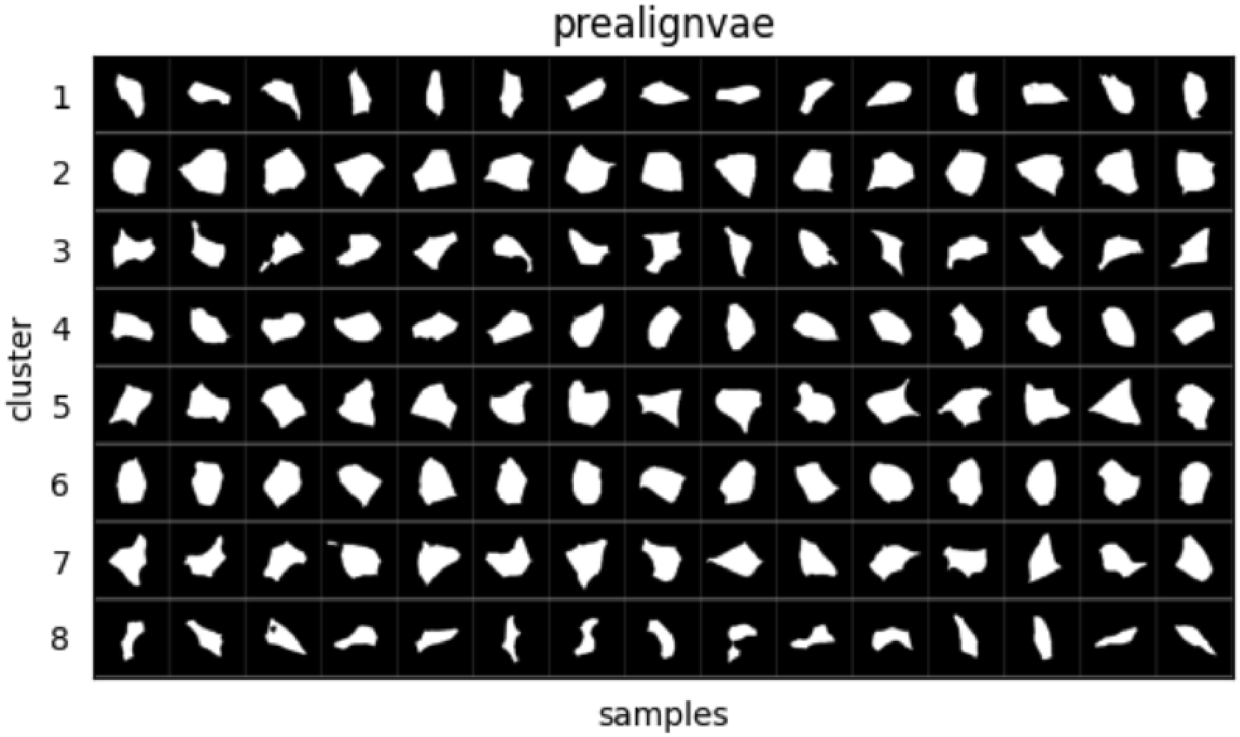

Supplementary Fig 18: Allen hiPSC scaled cells - clustering of prealign-VAE representations. GMM clustering over VAE representations with k=8 for hiPSC cells.

Commentary

- Orientation is not a confounder, and the shapes are reasonable.
- Prealign-vae also gives reasonable clusterings. (However recall that in the manuscript Results section 2, “Image pre-alignment fails to enforce orientation-invariant embedding spaces” we show that prealign-VAE has more errors on the real Allen dataset.)

Supplementary Note 2g: comparison of reconstructions of O2-VAE vs autoencoder baselines

In Fig.3b we propose some tests to verify that the learned representations by the O2-VAE is reasonable. One of them is reconstructions. In the next figure we compare O2-VAE reconstructions to VAE reconstructions.

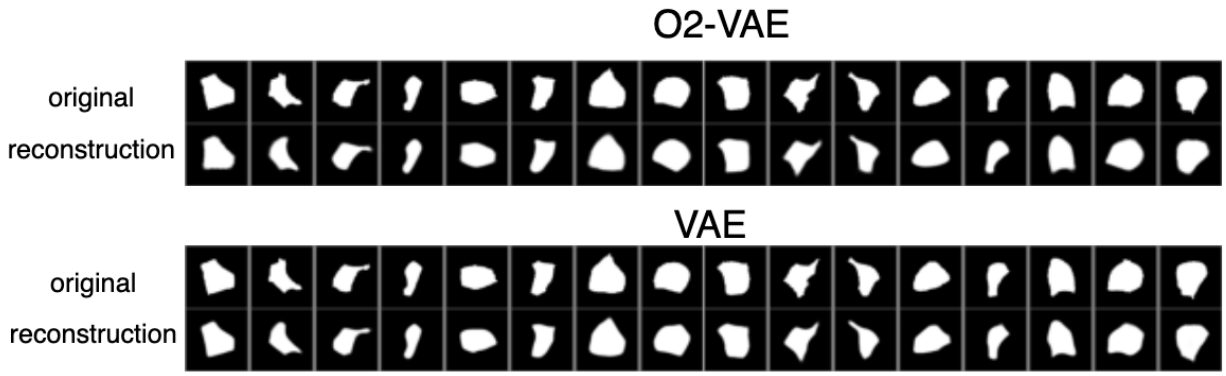

Supplementary Fig 19: reconstructions of samples from Allen hiPSC scaled cell dataset. Top: O2-VAE results. Bottom: VAE results.

Our experiments show that reconstruction quality is similar; VAE reconstructions are arguably a little bit sharper at the corners of the shape. But we argue that this is a secondary evaluation method: our goal is to learn good representations (as measured by clustering accuracy, linear probing), and we only require that reconstructions are ‘good enough’ to show the major image details for use in interpretability studies. Furthermore, machine learning literature on generative models argues that reconstruction quality is not necessarily correlated with representation quality [16].

Supplementary Note 2h: clustering errors due to orientation when using a VAE on a synthetic dataset.

We design a simple test to show that orientation affects the clustering accuracy. First we take the “Profiling Cell Shape and Texture (PCST)” dataset described in the text, which has 2 factors of variation: eccentricity and contour randomness, as illustrated by the next figure.

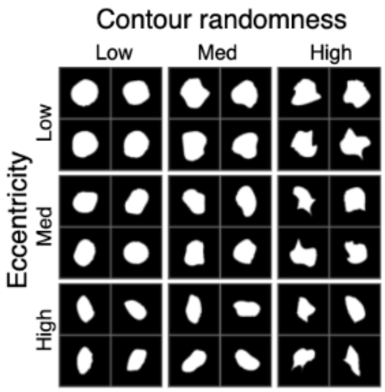

Supplementary Fig 20: samples from our synthetic cellular shape dataset. Varying eccentricity (columns) and contour randomness (rows), for 9 classes.

The test is:

- Create a second dataset where each image is randomly rotated by an angle sampled uniformly in (0,180°).
- Compute embeddings using either VAE or O2-VAE.
- Do clustering. We choose KMeans with k=10.
- If the cell and its rotated version are not in the same cluster, it is a 'clustering error'.

The average error for VAE is 58.5%, and for O2-VAE it is 7.4%. Note that the O2-VAE error is not exactly 0 because there are discretization errors introduced by the random rotation applied to the data, as well as discretization in the encoder (perfect continuous invariance is not possible).

In the next figure, we show how the error rates can vary based on dataset properties. In particular, more circular cells (with lower eccentricity) have lower error. The error is also lower when the angle is close to 0 or 180.

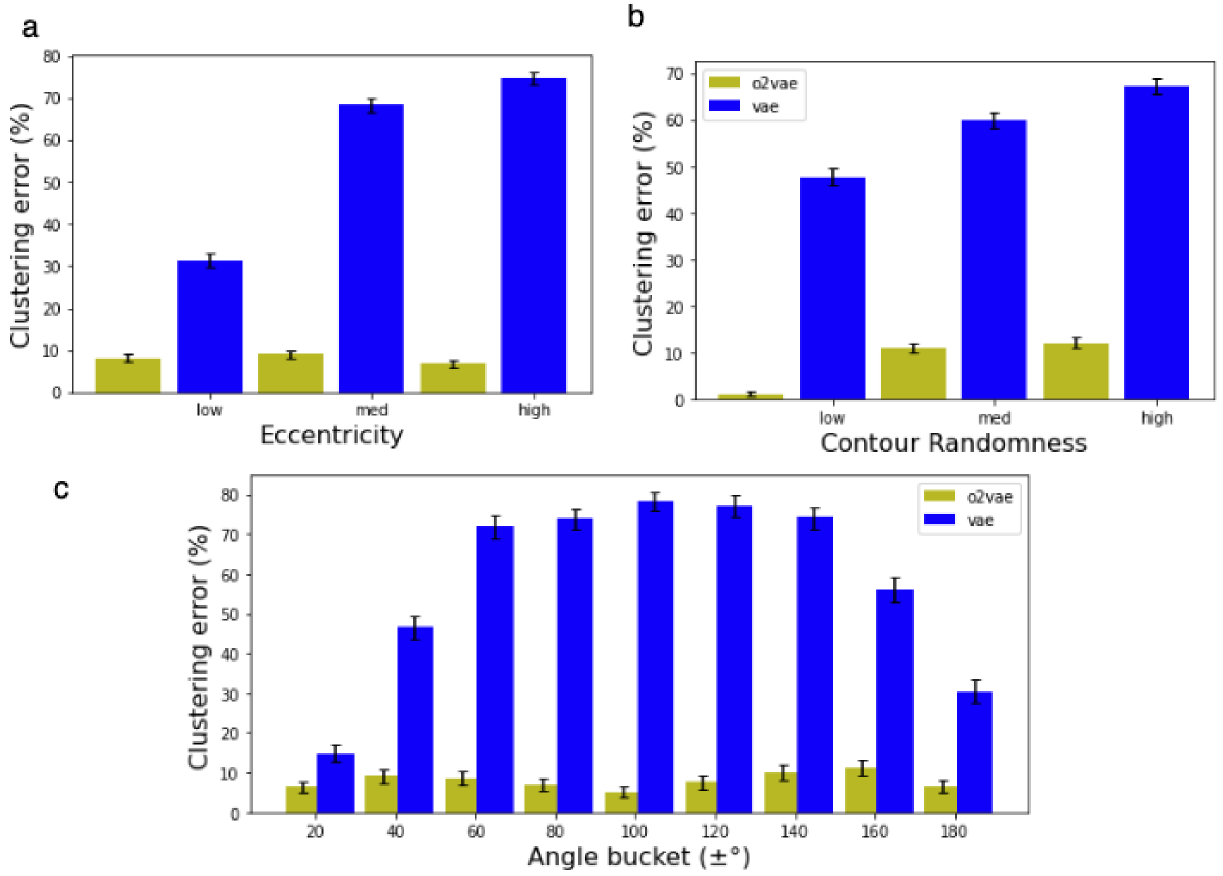

Supplementary Fig 21: error rates for cluster consistency as a function of (a) cell eccentricity, (b) cell contour randomness, and (c) angle of rotation. The dataset is visualised in Supplementary Fig.20. The 95% confidence intervals for the clustering error were computed using nonparametric bootstrapped resampling with 1000 repeats.

Supplementary Note 2i: embedding error and cluster error results from Fig.2 on a per-group basis

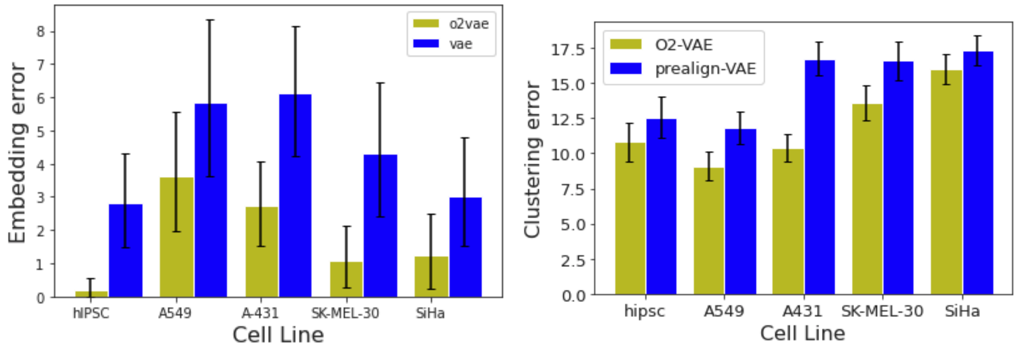

Supplementary Fig 22: In the main text, Fig 2.f shows embedding errors and clustering errors for hIPSC [1] and HPA [17]. Here we show the same plots (embedding error left, clustering error right), but with each HPA group summarised separately with its own 95% confidence intervals that were computed with nonparametric bootstrap.

Supplementary Note 3

This supplementary supports the fourth Results section.

Supplementary Note 3a: MEFs nucleus clustering

We show the clustering results for nuclei in MEFs to support the claim that LMNA deficient cells have lower prevalence of circular shape groups

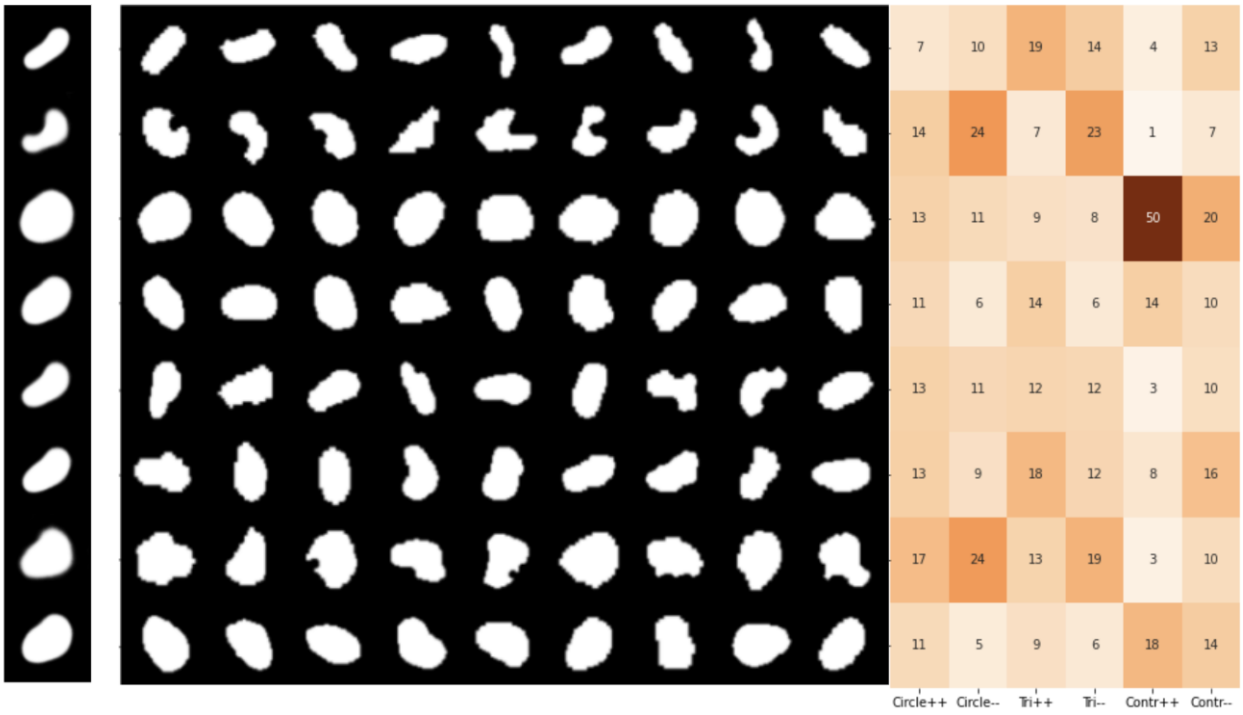

Supplementary Fig 23: clustering of MEFs nuclei
Nucleus clustering with GMM and k=8. Prototypes (left), cluster samples (middle) and population frequencies (right).

Supplementary Note 3b: Unsupervised mitosis detection in hiPSCs

Using the simple approach described in Results we show further details of the unsupervised approach for mitosis detection.

Stage	# samples	Accuracy
Interphase	9672	99.37%
Prophase	99	42.42%
Early prometaphase	82	100.00%
Prometaphase / metaphase	216	99.54%
Anaphase / telophase paired	63	100.00%
Anaphase / telophase unpaired	135	94.81%

Supplementary Table 1: class-level scores for unsupervised mitosis detection. For each mitosis state, accuracy scores for whether the cells in that state were detected as 'in mitosis' under the unsupervised detection model described in Results.

In Supplementary Table 1, half of the prophase states are missed, but all other states are discovered with more than 90% accuracy. We go deeper into these results with examples in the next figure

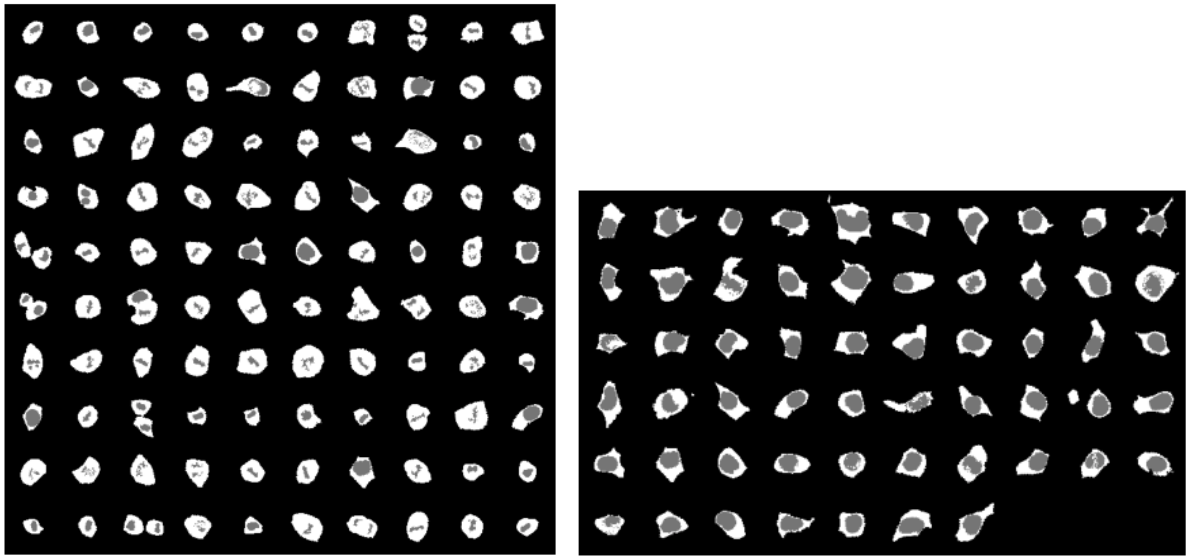

Supplementary Fig 24: prophase classes detected correctly and incorrectly
(left) Sample prophase cells that were correctly classified as "in mitosis" by our approach. (Right) prophase cells incorrectly classified as "normal" by our model. (the ground truth labels were generated by 3d regular images).

Note, in Supplementary Fig.24, that most of the prophase cells incorrectly labelled as normal by our approach really do look normal - many look like interphase cells. Their prophase

classification is likely based on either more details from 3D images, more details from grayscale information, or the given label could be an error.

Finally, our method labels some cells as outliers (mitosis) that are labelled as ‘normal’ in the Allen cell collection. We show what these cells look like in Supplementary Fig.25, and suggest that some of them *may* be mitosis outliers .If some of these really are in mitosis, then these samples illustrate the potential for an unsupervised approach to be a secondary check on other methods.

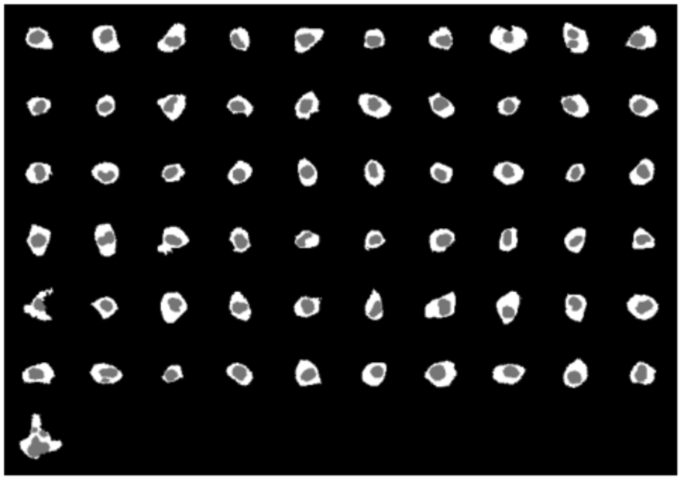

Supplementary Fig 25: candidate ‘missed’ mitosis cells. Sample cells that our unsupervised approach classified as ‘mitosis’ that were classified as normal in the dataset labels.

Supplementary Note 3c: Organelle contact rates

In the fourth results section and Fig.4e-f we report on contact rates for mitochondria subgroups. Here is some additional supporting data.

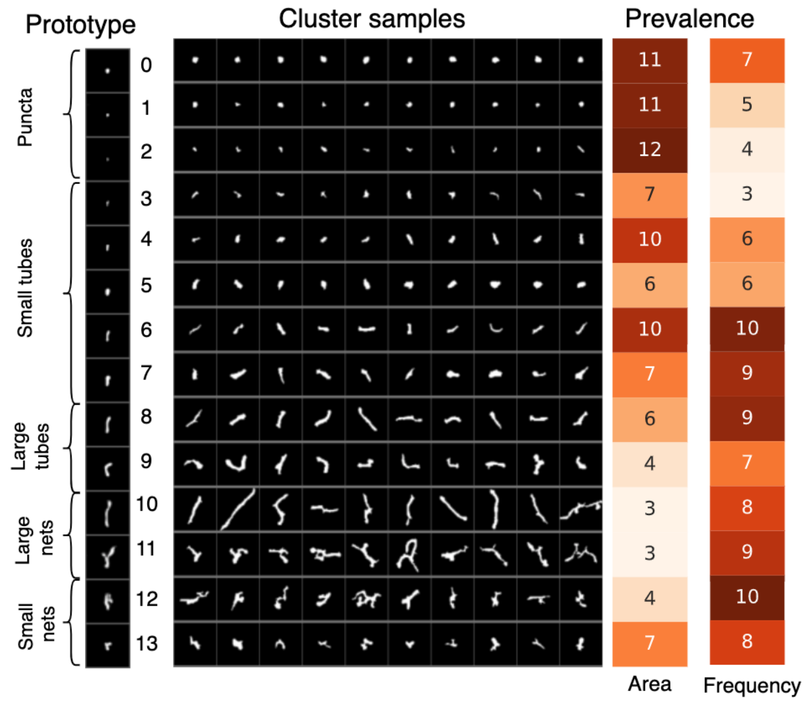

Supplementary Fig 26: cluster samples and prevalence. Prevalence are for mitochondria groups used in contact rate analysis.

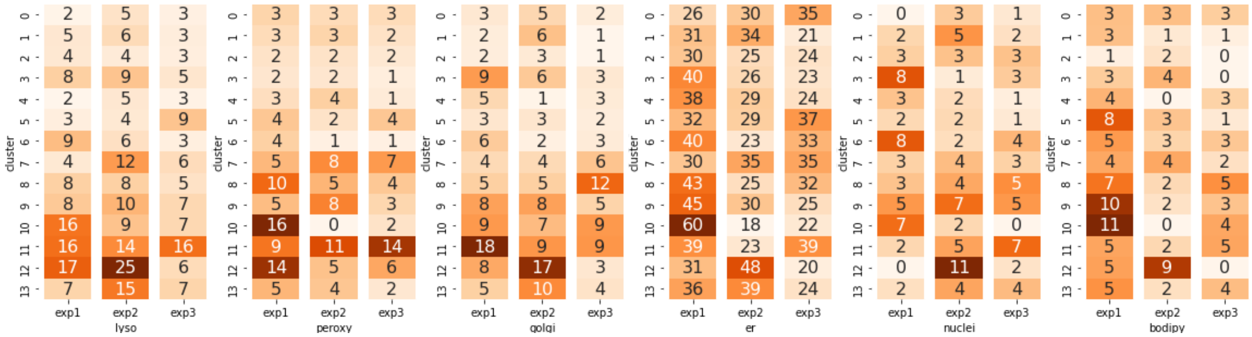

Supplementary Fig 27: contact rates from Fig.4g for sub-experiments. The sub-experiments from Fig.4g, broken down into 3 subgroups collected as different experiments: 'exp1', 'exp2', and 'exp3'. The last heatmap, 'bodipy' , is the tag for lipid droplet.

Supplementary Note 3d: clustering performance of o2vae vs baseline outline-PCA (using VAMPIRE) on MEFs dataset

In the main section Fig.4a we did shape clustering of the MEFs dataset, and identified clusters that had spikes, which are possibly filopodial. We identified this grouping by observing clusters with the following properties:

1. The 'cluster prototypes' had blurred edges, corresponding to an 'averaging' over many objects with spikes
2. A high portion of the samples from those clusters had spiked samples.

The prior work, VAMPIRE [2] did not identify these subgroups. Here, we reproduce VAMPIRE's analysis. We ran VAMPIRE's code [2] on the MEFs segmentations (the segmentation is described in Methods) with the default parameters. Supplementary Fig.28 shows the default outputs of the model. It has cluster prototypes (similar to ours), and cluster samples; their sampling visualisation is to superimpose the cell outlines. In Supplementary Fig 29, we show the same prototypes, but use our visualisation method, which is to cluster samples in a grid. We have rescaled the objects to have equal major axis length, which is in line with VAMPIRE's methodology.

We claim that, unlike in Fig.4a, Supp Figs.3f-g do not show clusters with the properties that we listed above:

1. The prototypes do not give any indication of spike shapes. It is interesting to note that since the O2VAE prototypes are represented as images, they can have 'fuzzy' boundaries to indicate higher variability around the border, whereas the VAMPIRE prototypes cannot because they are outlines.
2. We do not see groups where most samples have spikes. We instead see spiked samples appearing in multiple groups.

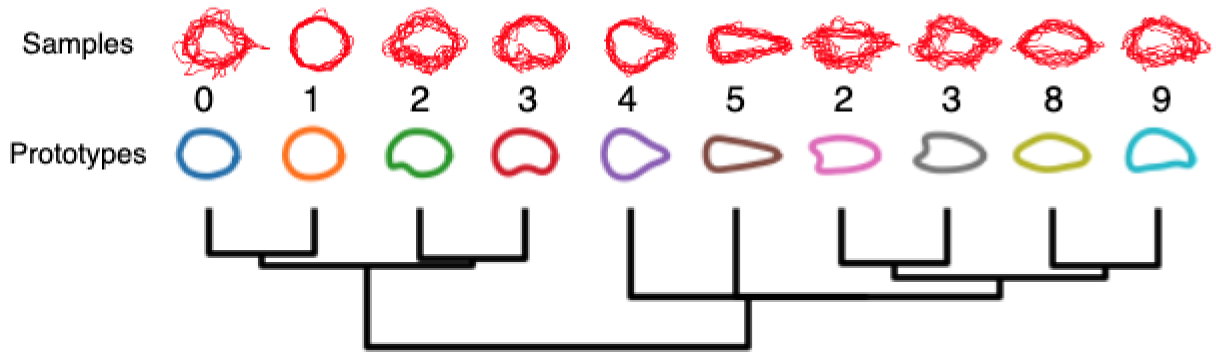

Supplementary Fig 28: MEFs cells - superimposed clustering samples and prototypes. These are the results from the VAMPIRE [2] paper.

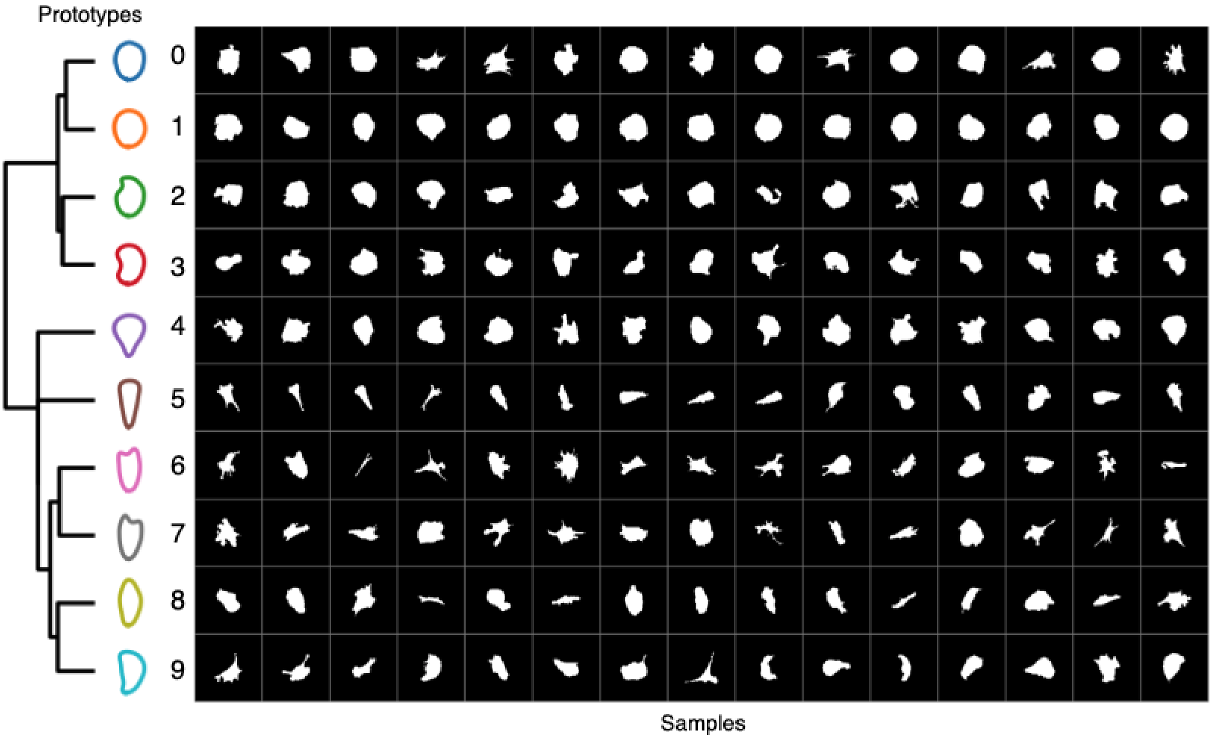

Supplementary Fig 29: MEFs cells - clustering sample images and prototypes. These results are derived from running the VAMPIRE [2] tool on this dataset.

Supplementary Note 3e: Clustering performance of o2vae vs baseline autoencoders on MEFs dataset

We repeat the clustering analysis from Figure.3a. The dataset is MEFs [2], which are scale-normalised based on major axis length. We do GMM clustering with K=10. Each row shows random samples from each cluster in their original orientation since orientation can be a confounder.

The conclusion from the three figures is that VAEs representations tend to group based on orientation and shape, while O2-VAE and prealign-VAE cluster based on shape.

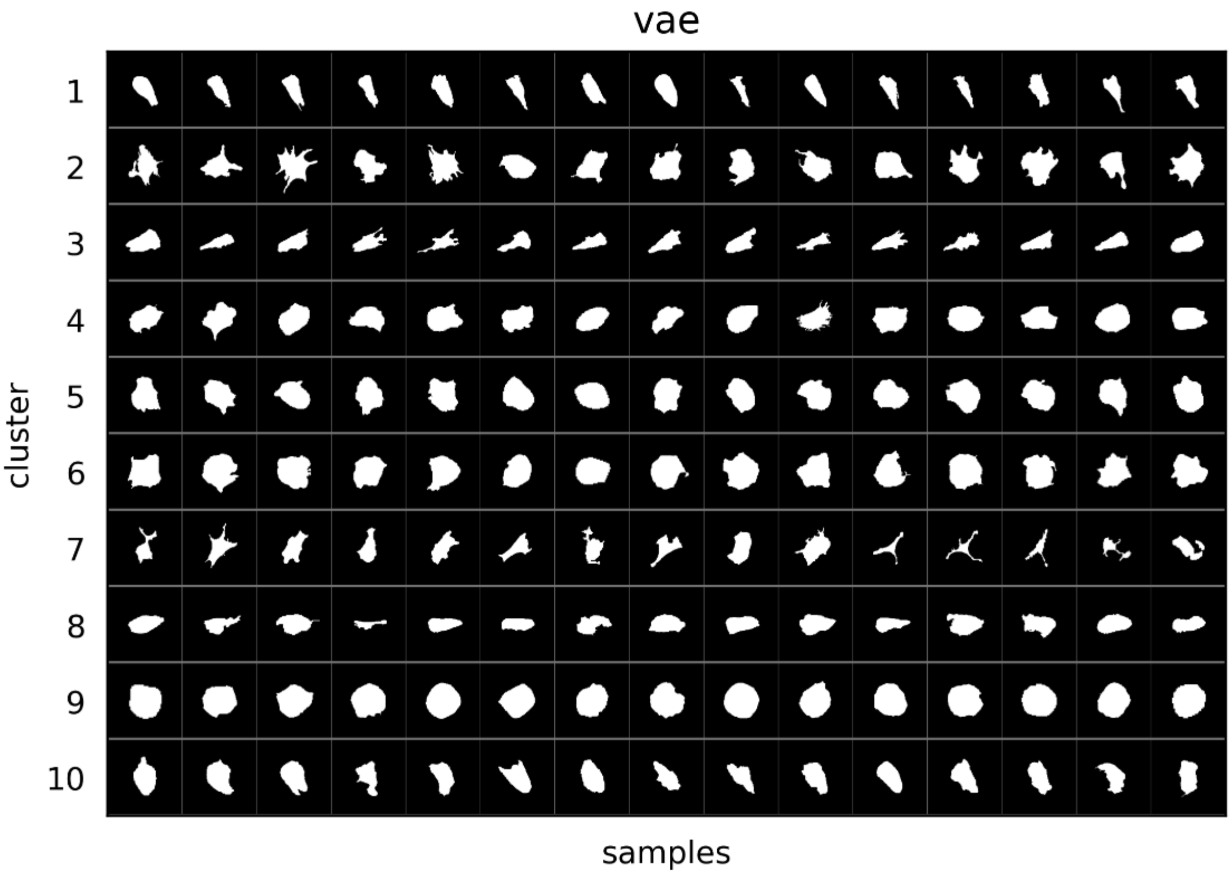

Supplementary Fig 30: MEFs cells - clustering of VAE representations

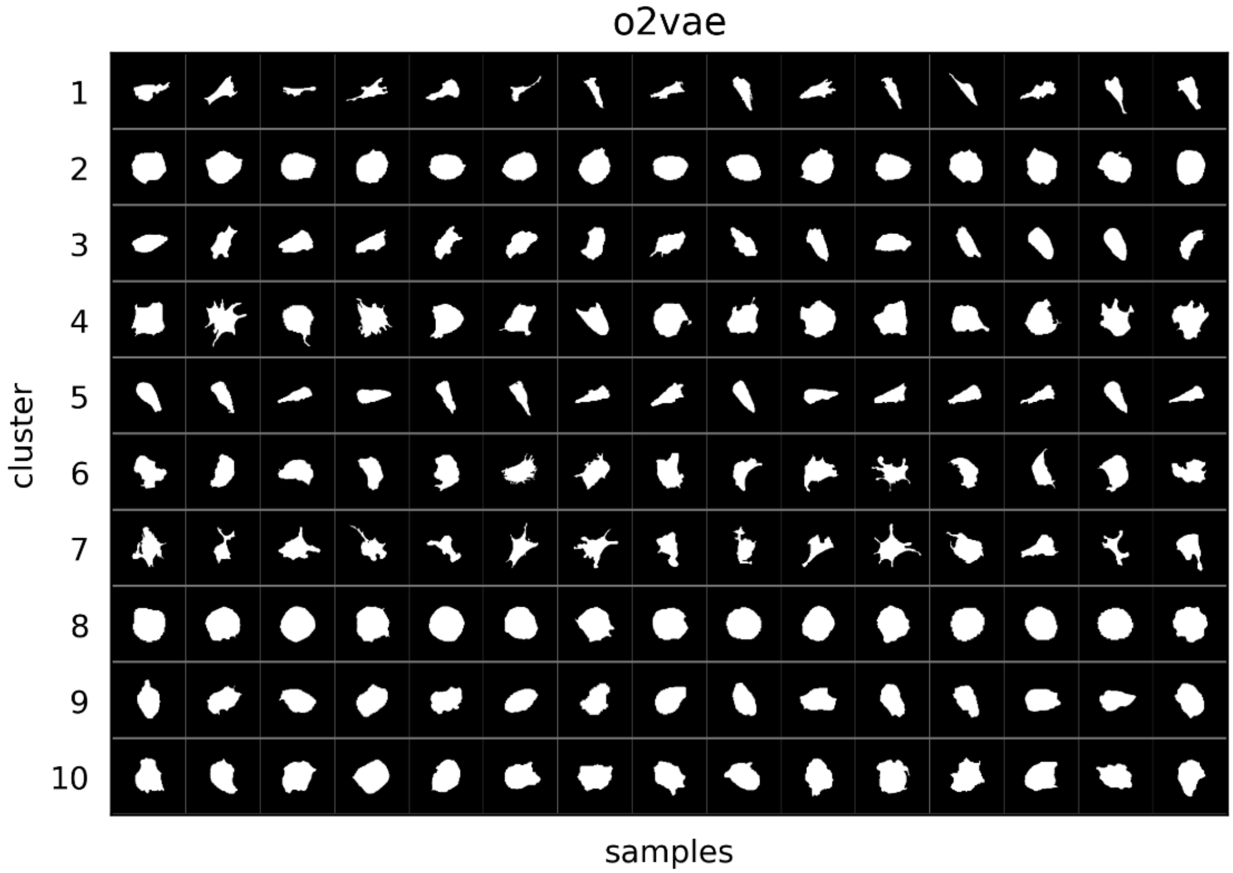

Supplementary Fig 31: MEFs cells - clustering of o2VAE representations

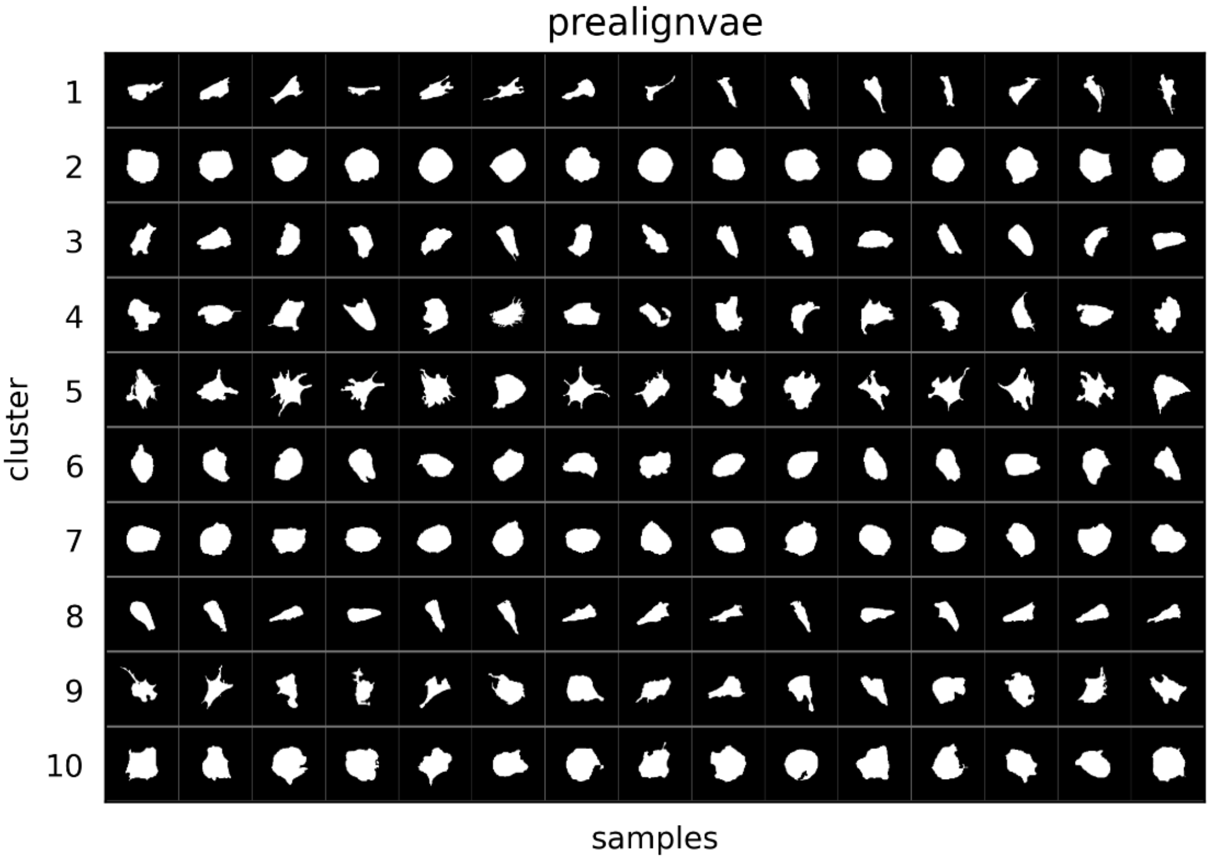

Supplementary Fig 32: MEFs cells - clustering of prealign-VAE representations

Supplementary Note 4

This supplementary supports the fifth Results section.

Supplementary Note 4a: Texture only experiments

In Supplementary Fig 33, we do the UMAPs for embeddings in the “texture only” experiment. **a**

b

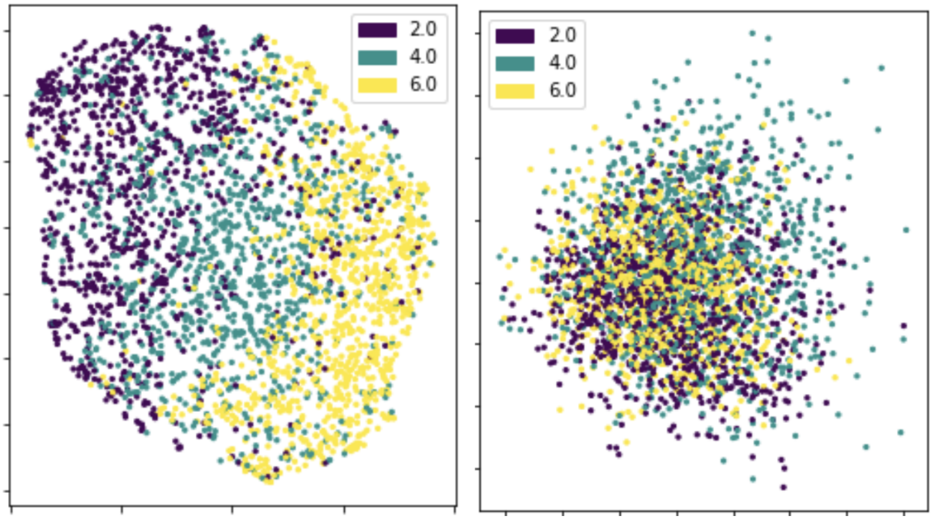

Supplementary Fig 33: dimensionality reduction of texture only experiment
a 2D UMAP reduction of data trained on a synthetic cell shape dataset with consistent shape, and varying texture, coloured by texture level. **b** PCA reduction (PC1 vs PC2) also coloured by texture.

Interestingly, though the UMAP approximately organises the embedding space by texture, the PC1-PC2 plot does not, so the texture variation in embedding space is not linear (we also checked that plotting lower PCs does not reveal the structure). But we do know that the classes really are separated based on the linear probing scores (0.93).

Supplementary Note 4b: Joint texture and shape experiments

Here we vary eccentricity, randomness, and texture (3 levels for each variable for 27 total classes). This extends Fig 5 where we did not model cell contour randomness. Generally we find that separating the classes with this many factors of variation becomes difficult (especially for texture).

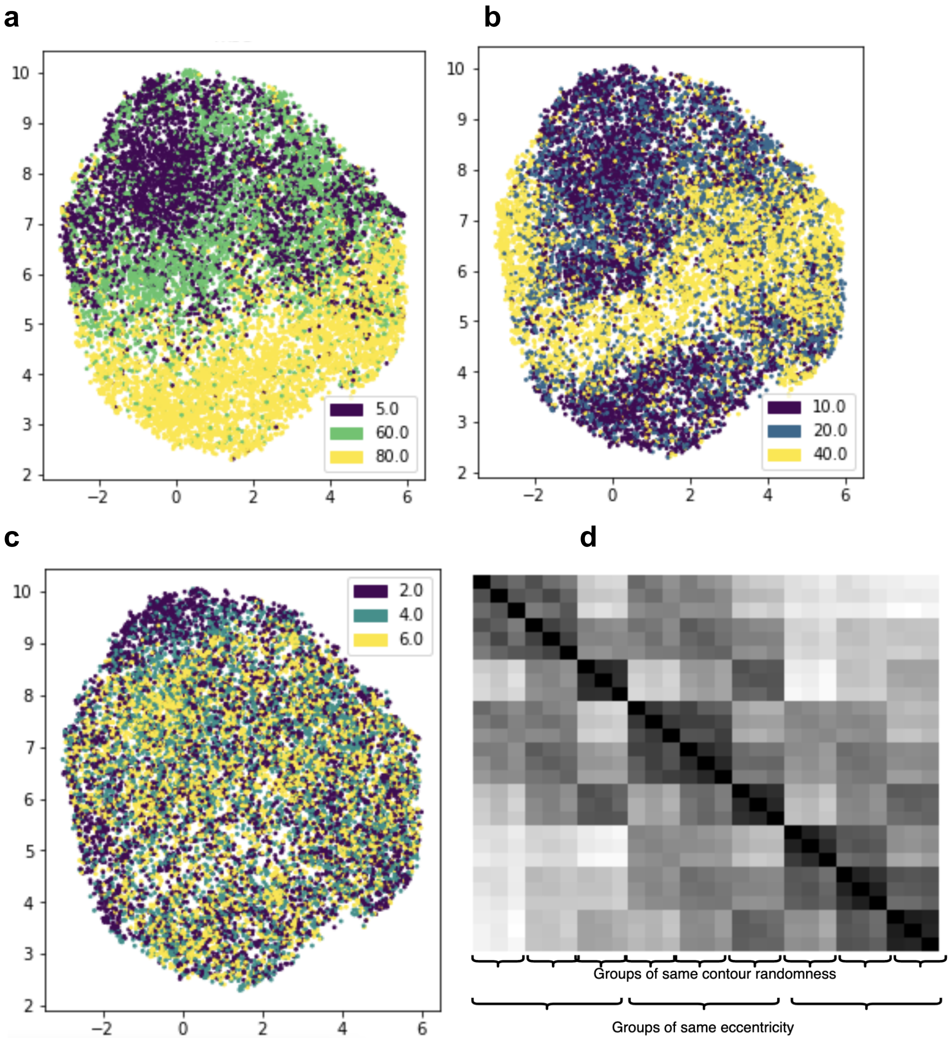

Supplementary Fig 34: summary representation space visualisations for joint shape and texture experiment. For the synthetic cell shape dataset, UMAP reductions are colored by **a** eccentricity, **b** contour randomness, and **c** texture. **d** distance matrix between the class centroids. There is a 3-level hierarchy of classes. The lowest is texture, so the first 3 classes are (low, medium, high) texture. The mid-level is eccentricity, so the first 3 cells are low contour randomness, then the next 3 are medium contour randomness and so on. The highest level is eccentricity.

First, these results show that there is a hierarchy for which factors of variation vary the most in representation space: eccentricity is the most dominant, then contour randomness, then texture. Second, texture features are not as well-separated in embedding space compared with the shape features, suggesting room for improving the model.

Supplementary Note 5

This supplementary supports the Discussion section.

Supplementary Note 5a: quantitative comparison of O2-VAE against alternative profiling methods, including autoencoders, contrastive methods, and engineered features

We quantitatively compare O2-VAE against other autoencoder methods, as well as other, non-autoencoder baselines: SimCLR [3] and Cellprofiler [4]. Note that ‘Cellprofiler’ is engineered features, and we use only those features that are orientation-invariant by construction.

Datasets

We use synthetic datasets because we can control the ground truth classes. The manuscript’s Methods section describes how they’re created. To briefly review, we use Simucell [5] to create cells with 3 factors of variation:

- Eccentricity (shape)
- Contour randomness (shape)
- Perlin texture (texture)

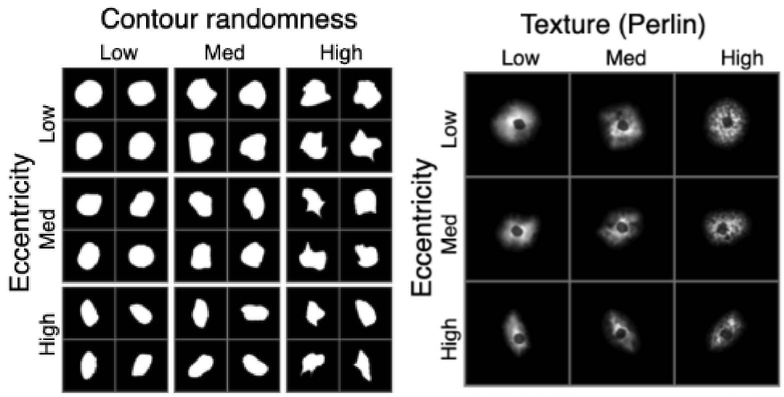

Supplementary Fig 35: data samples from our synthetic dataset. Left panel (main text Fig.3a) shows samples that vary in eccentricity and contour randomness. Right: (main text Fig.5a) shows samples that vary in eccentricity and perlin texture.

Using these factors of variation, we define the datasets:

- ‘Simucell_shape’: varying ‘eccentricity’ and ‘contour randomness’ (shape only).
- ‘Simucell_texture_eccentricity’: varying ‘eccentricity’ and ‘texture’ (texture & shape).
- Simucell_texture_and_shape: varying all factors, ‘eccentricity’, ‘contour randomness’ and ‘texture’ (texture & shape).

We also use a real dataset, ‘nucleus_mitosis’:

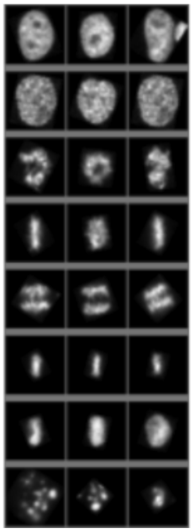

Supplementary Fig 36: data samples from CellCognition [6]. This is a real dataset of textured nuclei undergoing mitosis, where the ground truth labels are the mitosis state, as labelled. This is called ‘nucleus_mitosis’.

Metrics

We do two tests, which are both described in the manuscript Methods section in more detail. Briefly, they are:

- Linear probing. We split the dataset into train and test sets. Using the train set, we use ground truth labels to train a classifier. We then evaluate classifier accuracy on the test set. This measures (i) how well-separated the embedding space is, and (ii) the utility of representations on downstream supervised learning tasks.
- Clustering accuracy. We do GMM clustering, where we assume that we know the correct number of clusters. We use Hungarian matching [7] to match each cluster with a ground truth class, and then compute the accuracy.

The synthetic Simucell datasets are balanced, so we report average accuracy. The ‘nucleus_mitosis’ dataset is imbalanced, so we use the ‘macro average’: we compute each class average separately, then take the mean of those numbers.

Models in comparison

As in the paper, we compare our O2-VAE method to VAE and prealign-VAE. As suggested by reviewers 1 and 3, we add two new baselines:

- SimCLR is a contrastive learning method [3]. We train with the same hyperparameters used in [8].
- CellProfiler features. We compute every possible feature and, as recommended by [9], we do z-score normalization. We try two sets of features: (i) using all features, and (ii) using only O2-invariant features. The latter, which we call ‘cellprofiler-o2’ had slightly better scores in most tests, so we report results from that model. We determine which features are invariant by computing the all metrics with the original image, and the image after a 90° rotation, and checking that the difference is smaller than some tolerance

Results for shape datasets (quantitative)

We first report results for shape-only datasets. Supplementary Table 1 shows the metrics. Supplementary Table 2 shows the *difference* in scores between O2-vae in that model. For example in Supplementary Table 2, we see that for dataset 'simucell_shape' and factor 'contour_random', O2-VAE has 9.8% higher linear probing score than VAE.

**Linear probing accuray**						
**dataset**	**factor**	**o2vae**	**vae**	**prealignvae**	**simclr**	**cellprofiler-o2**
simucell_shape	eccentricity	91.5%	89.3%	90.4%	89.5%	91.8%
simucell_shape	contour_random	88.9%	79.1%	87.2%	84.4%	89.3%
simucell_shape	all	82.6%	73.0%	82.0%	76.7%	83.4%
**Clustering accuracy**						
**dataset**	**factor**	**o2vae**	**vae**	**prealignvae**	**simclr**	**cellprofiler-o2**
simucell_shape	eccentricity	85.1%	52.4%	87.1%	59.1%	63.8%
simucell_shape	contour_random	37.1%	41.7%	38.9%	47.6%	52.6%
simucell_shape	all	47.5%	37.6%	49.4%	43.8%	61.9%

Supplementary Table 2: quantitative metrics comparison for synthetic shape datasets

**Linear probing accuracy- difference with O2VAE, Shape**					
**dataset**	**factor**	**vae**	**prealignvae**	**simclr**	**cellprofiler-o2**
simucell_shape	eccentricity	2.2%	1.0%	2.0%	-0.3%
simucell_shape	contour_random	9.8%	1.7%	4.4%	-0.4%
simucell_shape	all	9.6%	0.6%	5.9%	-0.8%
**Clustering accuracy - difference with O2VAE**					
**dataset**	**factor**	**vae**	**prealignvae**	**simclr**	**cellprofiler-o2**
simucell_shape	eccentricity	32.8%	-2.0%	26.0%	21.3%
simucell_shape	contour_random	-4.6%	-1.9%	-10.5%	-15.5%
simucell_shape	all	9.9%	-1.9%	3.7%	-14.4%

Supplementary Table 3: quantitative metrics showing relative change of various methods against O2VAE for synthetic shape datasets.

Summary for linear probing:

- O2-VAE vs VAE: O2-VAE is consistently better.
- O2VAE vs Prealign-VAE: similar scores (However recall that in the manuscript Results section, "Image pre-alignment fails to enforce orientation-invariant embedding spaces" we show that prealign-VAE has more errors on the real Allen dataset.)
- O2VAE vs SimCLR: O2-VAE is slightly better.
- O2VAE vs Cellprofiler: similar.

Summary for clustering accuracy:

- In clustering, we have no supervision, so it is impossible to get good scores for clustering eccentricity *and* contour randomness simultaneously (since the algorithm's clustering will be the same for both tests).
- For eccentricity: the O2VAE and prealignVAE methods have high scores (>80%), while Simclr and Cellprofiler score at ~60%.
- For contour randomness: no methods perform well, but SimClr and cellprofiler perform slightly better. (about 50%).

Results for texture datasets (quantitative)

Similar to the last section, Table 3 shows metrics for texture datasets, and Table 4 shows the difference of models compared to O2VAE. For example in Table 4, in the dataset 'simucell_texture_eccentricity' and factor 'texture', O2VAE has 27.7% higher linear probing score than VAE.

**Linear probing accuracy**						
**dataset**	**factor**	**o2vae**	**vae**	**prealignvae**	**simclr**	**cellprofiler-o2**
simucell_texture_eccentricity	eccentricity	99.6%	97.6%	99.1%	97.5%	99.3%
simucell_texture_eccentricity	texture	79.8%	52.1%	58.4%	87.7%	95.3%
simucell_texture_eccentricity	all	81.4%	50.4%	60.7%	85.5%	93.6%
simucell_texture_and_shape	eccentricity	87.5%	84.9%	86.8%	84.3%	87.6%
simucell_texture_and_shape	contour_random	83.7%	72.6%	80.2%	69.1%	85.3%
simucell_texture_and_shape	texture	53.3%	43.0%	51.4%	76.1%	92.4%
simucell_texture_and_shape	all	43.9%	26.4%	35.8%	42.5%	70.7%
nucleus_mitosis	all	74.8%	62.3%	82.8%	90.1%	90.1%
**Clustering accuracy**						
**dataset**	**factor**	**o2vae**	**vae**	**prealignvae**	**simclr**	**cellprofiler-o2**
simucell_texture_eccentricity	eccentricity	69.10%	65.64%	97.86%	51.77%	98.52%
simucell_texture_eccentricity	texture	34.18%	33.86%	34.19%	44.11%	33.66%
simucell_texture_eccentricity	all	31.64%	29.41%	33.47%	24.51%	56.92%
simucell_texture_and_shape	eccentricity	58.15%	59.04%	84.46%	58.33%	64.96%
simucell_texture_and_shape	contour_random	41.27%	34.19%	37.43%	34.45%	44.11%
simucell_texture_and_shape	texture	34.17%	33.88%	33.99%	42.02%	46.67%
simucell_texture_and_shape	all	16.90%	13.72%	17.92%	13.51%	32.93%
nucleus_mitosis	all	42.28%	32.77%	48.81%	38.71%	46.53%

Supplementary Table 4: quantitative metrics comparison for synthetic texture datasets.

Linear probing - difference with O2VAE					
**dataset**	**factor**	**vae**	**prealignvae**	**simclr**	**cellprofiler-o2**
simucell_texture_eccentricity	eccentricity	1.9%	0.4%	2.1%	0.3%
simucell_texture_eccentricity	texture	27.7%	21.4%	-7.9%	-15.5%
simucell_texture_eccentricity	all	31.0%	20.7%	-4.1%	-12.2%
simucell_texture_and_shape	eccentricity	2.5%	0.7%	3.1%	-0.1%
simucell_texture_and_shape	contour_random	11.1%	3.5%	14.6%	-1.6%
simucell_texture_and_shape	texture	10.4%	1.9%	-22.8%	-39.1%
simucell_texture_and_shape	all	17.5%	8.1%	1.4%	-26.8%
nucleus_mitosis	all	12.6%	-7.9%	-15.2%	-15.2%
**Clustering accuracy - difference with O2VAE**					
**dataset**	**factor**	**vae**	**prealignvae**	**simclr**	**cellprofiler-o2**
simucell_texture_eccentricity	eccentricity	3.46%	-28.76%	17.33%	-29.42%
simucell_texture_eccentricity	texture	0.32%	-0.01%	-9.93%	0.52%
simucell_texture_eccentricity	all	2.23%	-1.82%	7.13%	-25.28%
simucell_texture_and_shape	eccentricity	-0.90%	-26.31%	-0.19%	-6.81%
simucell_texture_and_shape	contour_random	7.08%	3.84%	6.81%	-2.84%
simucell_texture_and_shape	texture	0.29%	0.18%	-7.85%	-12.50%
simucell_texture_and_shape	all	3.18%	-1.02%	3.39%	-16.04%
nucleus_mitosis	all	9.50%	-6.53%	3.56%	-4.26%

Supplementary Table 5: quantitative metrics showing relative change of various methods against O2VAE for synthetic texture datasets.

Summary for linear probing:

- O2VAE vs VAE: O2-VAE is better in all cases.
- O2VAE vs Prealign-VAE: O2-VAE is better in all cases with one exception.
- O2VAE vs SimCLR: O2-VAE is slightly better for shape, but substantially worse for texture.
- O2VAE vs CellProfiler: O2-VAE is similar for shape, but substantially worse for texture.
- compared to SimClr and Cellprofiler, is slightly better or equal when measuring howe

Summary for clustering:

- As before, it is impossible to get good clustering scores on all factors simultaneously. The results are very mixed. For these datasets, almost all scores are below 50%, so the performance is poor. The exception is that for `simucell_texture_eccentricity` (varying eccentricity and texture), the eccentricity is well clustered by Cellprofiler, and to a lesser extent, by O2VAE and PrealignVAE.

Discussion on the results

For shape datasets, these show that our O2-VAE outperforms autoencoder baselines. For other baselines, namely SimCLR [3] and Cellprofiler [4], the performance is similar: O2VAE better for some datasets and worse for others. O2VAE shows good performance for morphology and shape-based tasks, which were the main tasks driving the development of our method.

For texture datasets, O2-VAE has better performance than autoencoder baselines, but worse performance than SimCLR and CellProfiler. However, we expect that O2-VAE texture representations can be improved in future work. This is because, for simplicity, we implemented a convolution VAE architecture with the standard loss function [10], which is known to poorly generate texture details [11, 12]. Machine learning literature shows that this can be improved with modifications to the autoencoder, for example with the VQ-VAE architecture [13, 14]. One prior work, Cytoself, has already demonstrated that the VQ-VAE architecture can model textured biomedical data (in the self-supervised setting) [15].

Supplementary Note 5b: orientation dependence of the Cytoself profiling model

Overview

The Cytoself model [15] is a recent method for cell profiling with an autoencoder. Unlike O2-VAE, it is trained with some supervision (marker prediction). As discussed in the Main Discussion section, orientation-as-a-confounder is likely less of an issue for this data. Here, we show that the model still has some dependence on orientation that impacts downstream task performance.

Invariance test

We design a very simple test of orientation-dependence.

- We download a model checkpoint from the cytoself repository [15], and a subset of the cytoself dataset (74249 images).
- We compute a representation for each image, z_i, and a second representation, z_i`, for the image after a 90° rotation:

- 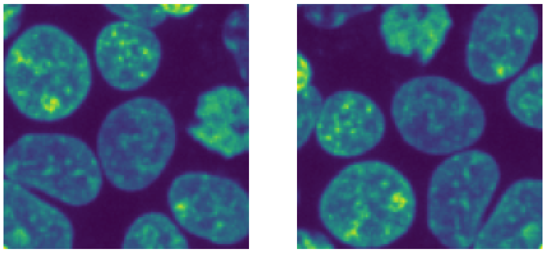
    - By choosing only a 90° rotation, we know that there are no artifacts in the image due to image transformations. Any analysis should perform the same when using z_i and z_i`.
- We train two models. They are trained to predict the labeled localization compartment: one of 'ER', 'Golgi', 'cytoplasm', 'mitochondria', 'nuclear domains', 'nuclear membrane', 'nucleolus', 'nucleoplasm', 'vesicles'. This is one of the groupings analysed in the Cytoself paper.
    - This is only nine classes, and so is likely an easier classification task than predicting protein, which has 1,311 classes.
- The first model is trained on the original images, and the second is trained on the 90° rotation images. If there is disagreement between the model predictions, that is an indicator of representation sensitivity.
- We train two models:
    - (i) Nearest-centroid: compute the L2-centroid of all embeddings in a class. New test samples are assigned to the class with the closest centroid.
    - (ii) logistic regression with L2 regularization implemented in scikit-learn.
- We report the rate of disagreement. We also report the rate of disagreement where the non-rotated model prediction is correct, which helps control for labeling errors.

	% disagreement	% disagreement where non-rotated model is correct
Nearest-centroid	7.8%	4.0%
Regression	3.6%	1.8%

- We repeated this analysis after first doing a UMAP dimensionality reduction (which is what the paper does), and found the rate of agreement was worse.

That experiment tested classification to localization compartments. The Cytoself paper also reported grouping of complexes, which are '20S', 'COPII', 'Clathrins', 'CoreRNAPol', 'Mediator', 'Nuclear pore', 'OST', 'Ribosome', 'SF3'. Here, the prediction model matched in 99.9% of samples, so orientation sensitivity was not an issue for this task.

Supplementary References

[1] Viana, M. P., Chen, J., Knijnenburg, T. A., Vasan, R., Yan, C., Arakaki, J. E., ... & Rafelski, S. M. (2020). Robust integrated intracellular organization of the human iPS cell: where, how much, and how variable. BioRxiv, 2020-12.

[2] Phillip, J. M., Han, K. S., Chen, W. C., Wirtz, D., & Wu, P. H. (2021). A robust unsupervised machine-learning method to quantify the morphological heterogeneity of cells and nuclei. Nature protocols, 16(2), 754-774.

[3] Chen, T., Kornblith, S., Norouzi, M., & Hinton, G. (2020, November). A simple framework for contrastive learning of visual representations. In International conference on machine learning (pp. 1597-1607). PMLR.

[4] Stirling, D. R., Swain-Bowden, M. J., Lucas, A. M., Carpenter, A. E., Cimini, B. A., & Goodman, A. (2021). CellProfiler 4: improvements in speed, utility and usability. BMC bioinformatics, 22, 1-11.

[5] Rajaram, S., Pavie, B., Hac, N. E., Altschuler, S. J., & Wu, L. F. (2012). SimuCell: a flexible framework for creating synthetic microscopy images. Nature methods, 9(7), 634-635.

[6] Held, M., Schmitz, M. H., Fischer, B., Walter, T., Neumann, B., Olma, M. H., ... & Gerlich, D. W. (2010). CellCognition: time-resolved phenotype annotation in high-throughput live cell imaging. Nature methods, 7(9), 747-754.

[7] Kuhn, H. W. (1955). The Hungarian method for the assignment problem. Naval research logistics quarterly, 2(1‐2), 83-97.

[8] Perakis, A., Gorji, A., Jain, S., Chaitanya, K., Rizza, S., & Konukoglu, E. (2021). Contrastive learning of single-cell phenotypic representations for treatment classification. In Machine Learning in Medical Imaging: 12th International Workshop, MLMI 2021, Held in Conjunction with MICCAI 2021, Strasbourg, France, September 27, 2021, Proceedings 12 (pp. 565-575). Springer International Publishing.

[9] Caicedo, J. C., Cooper, S., Heigwer, F., Warchal, S., Qiu, P., Molnar, C., ... & Carpenter, A. E. (2017). Data-analysis strategies for image-based cell profiling. Nature methods, 14(9), 849-863.

[10] Kingma, D. P., & Welling, M. (2013). Auto-encoding variational bayes. arXiv preprint arXiv:1312.6114.

[11] Kingma, D. P., & Welling, M. (2013). Auto-encoding variational bayes. arXiv preprint arXiv:1312.6114.

[12] Esser, P., Rombach, R., & Ommer, B. (2021). Taming transformers for high-resolution image synthesis. In Proceedings of the IEEE/CVF conference on computer vision and pattern recognition (pp. 12873-12883).

[13] Razavi, A., Van den Oord, A., & Vinyals, O. (2019). Generating diverse high-fidelity images with vq-vae-2. Advances in neural information processing systems, 32.

[14] Van Den Oord, A., & Vinyals, O. (2017). Neural discrete representation learning. Advances in neural information processing systems, 30.

[15] Kobayashi, H., Cheveralls, K. C., Leonetti, M. D., & Royer, L. A. (2022). Self-supervised deep learning encodes high-resolution features of protein subcellular localization. Nature methods, 19(8), 995-1003.

[16] Theis, L., Oord, A. V. D., & Bethge, M. (2015). A note on the evaluation of generative models. arXiv preprint arXiv:1511.01844.

[17] Thul, Peter J., et al. "A subcellular map of the human proteome." Science 356.6340 (2017): eaal3321.
